# Supplementary material for: A DNA Vaccine That Encodes an Antigen-Presenting Cell-Specific Heterodimeric Protein Protects against Cancer and Influenza
Source: Mol Ther Methods Clin Dev. 2020 Jan 25;17:378–92. doi: 10.1016/j.omtm.2020.01.007 (PMC7044496; doi:10.1016/j.omtm.2020.01.007)
Supplement: Document S1. Figures S1–S4 and Supplemental Materials and Methods [file mmc1.pdf]

OMTM, Volume *17*

Supplemental Information

A DNA Vaccine That Encodes an Antigen-Presenting Cell-Specific Heterodimeric Protein Protects against Cancer and Influenza

Ranveig Braathen, Heidi Cecilie Larsen Spång, Daniëla Maria Hinke, Jana Blazevski, Sonja Bobic, Even Fossum, and Bjarne Bogen

Supplemental information

Supplemental Figures

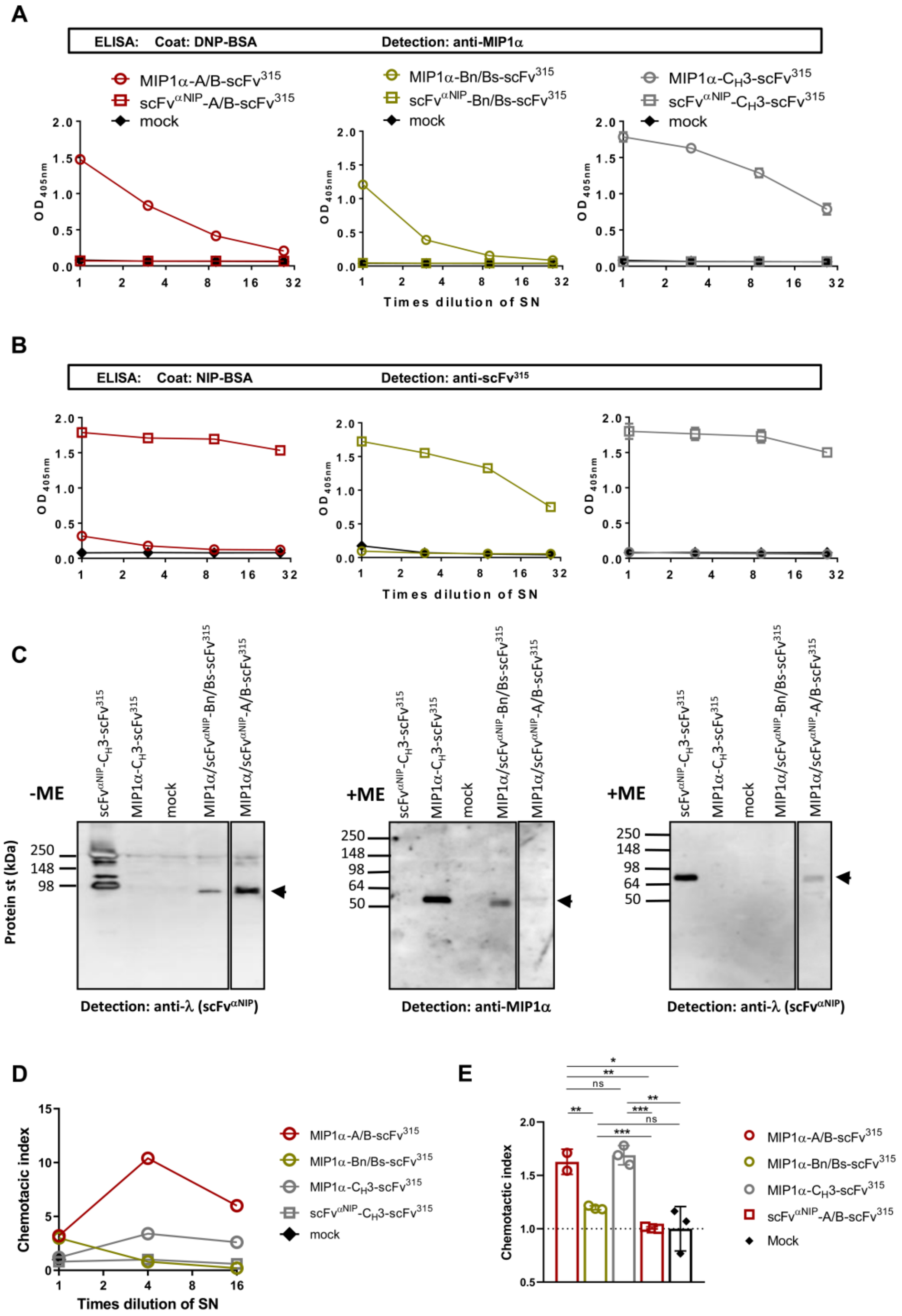

Figure S1. Characterisation of heterodimeric and homodimeric APC-targeting DNA vaccines. A-B and D. Supernatants from HEK293E cells transiently transfected with the indicated combinations of DNA plasmids were analyzed in the specified sandwich ELISAs (mean ±SD) (A-B) and for chemotaxis of $CCR1^+$ $CCR5^+$ ESb-MP cells (D). **C.** Western analysis of homodimeric and heterodimeric APC-targeting DNA vaccines. Western analysis were performed on supernatants from HEK293E cells transiently transfected with the indicated combinations of DNA plasmids containing a HIS tag. The supernatants were purified on a HIS-column before adding to the gel under non-reducing (-ME) or reducing (+ME) conditions and vaccine proteins detected as specified. An arrowhead shows the protein band detected by the specific antibodies against λ-chain in $scFv^{315}$ or MIP1α, respectively. The vertical lines indicate where parts of the image were joined. **E.** Chemotaxis on DC-enriched bone marrow cells from BALB/c mice was performed with 40 nM of the indicated affinity purified vaccine proteins (n=3 filter/vaccine group, n=2 for MIP1α-A/B-$scFv^{315}$, mean±SD, ns=non-significant, *p<0.05, **p<0.01 ***p<0.001, Unpaired T test).

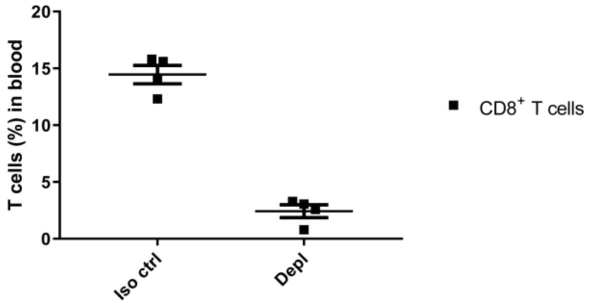

Figure S2. Depletion of CD8$^+$ T cells by injection of anti-CD8 mAb. Mice were immunized with 50 µg i.m./EP of MIP1α-A/B-scFv^{315} DNA vaccine. From day 12 mice were injected every other day for 14 days and after that once a week with 100 µg of depleting mAb against CD8 or with isotype control Ab. 2x10^5 MOPC315.BM.Luc tumor cells were used i.v. as challenge on day 14 after vaccination. Amount of T cells (%) in peripheral blood at day 38 after challenge in the specified group (n=4/group).

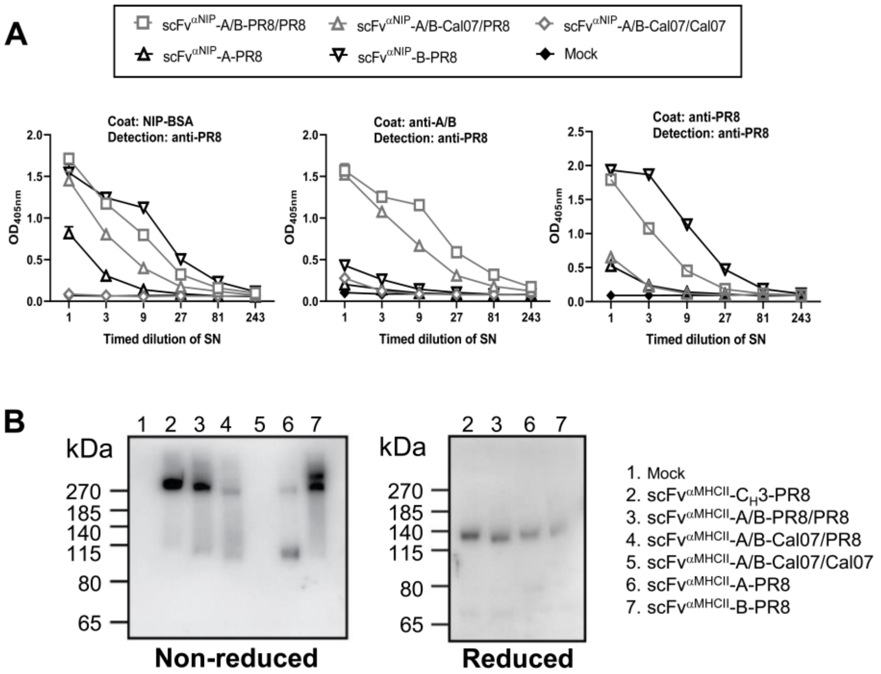

Figure S3. Both the ACID and BASE chains alone can be expressed in vitro. A. HEK293E cells were transiently transfected with a total amount of 5 μg of DNA per transfection with the indicated combinations of DNA plasmids. Supernatants were analyzed in the indicated sandwich ELISAs (mean of triplicates ±SD). The graph to the right indicates preferential A/B pairing since the signal caused by B/B dimer formation (PR8 on both arms, detected in the anti-PR8/anti PR8 sandwich ELISA) was attenuated by inclusion of an A-chain with a different antigen (Cal07 HA). **B**. Western blot analysis of supernatants. Supernatants were first normalized on dot blots. Adjusted amounts were loaded onto the gel under non-reducing (-DTT) or reducing (+0.1 M DTT) conditions. Vaccine proteins were detected with anti-PR8 specific mAb.

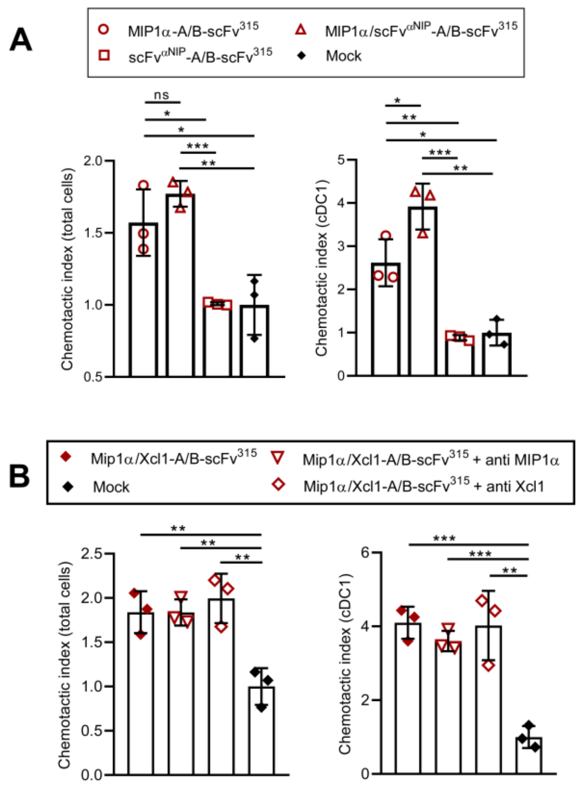

Figure S4. A single APC-specific targeting unit in the ACID/BASE heterodimeric vaccine is sufficient for chemotaxis of cDC1 cells. A. Chemotaxis on Flt3L induced DC-enriched bone marrow cells from BALB/c mice was performed with 40 nM of the indicated purified vaccine proteins. The chemotaxis index was measured by flow and shown for either total number of cells (left) or cDC1 ($CD11c^{+}CD45^{-}CD11b^{-}CD24^{+}$) cells (right). **B.** Purified vaccine protein (2 nM), with MIP1α chemokine on the A arm and Xcl-1 chemokine (a known cDC1 attractant) on the B arm, were tested for their ability to attract total cells (left) or cDC1 (right) in the presence of either no mAb, or anti-MIP1α mAb or anti-Xcl1 mAb in 40-fold molar excess (n=3 filter/vaccine group, mean±SD, ns=non-significant, **p<0.01 ***p<0.001, Unpaired T test).

Supplemental Methods

T cells in peripheral blood

Blood samples (50 µl) were collected from the saphenous vein into a tube precoated with 50µl of 1:10 Heparin: H20 solution (Heparin 5000 IE/ml). Cells were centrifuged (1400 rpm, 7min, 4°C) and incubated with ACT buffer on ice 5-10 min and washed 3x PBS+0.5% BSA. Cells were blocked for unspecific binding by the use of 30% rat serum for 20 min at 4°C. Subsequent staining was done with following antibodies, according to manufacturer`s recommendations: Anti-Mouse CD19- FITC (35-0193-U500, TONBO); Anti-Mouse CD14-FITC (553739, BD Pharmingen); Anti-Mouse CD3-VF450 (75-0032-U100, TONBO); Anti-Mouse CD8-AF700 (100730, Biolegend). The cells were analyzed on the Attune Nxt acoustic focusing cytometer (Life Technologies), using FlowJo software. Cells of interest were gated as $CD19^{-}CD14^{-}CD3^{+}CD8^{+}$.

Purification of vaccine proteins

For purification of $scFv^{315}$ containing vaccine proteins, $2x10^{7}$ HEK293E cells were seeded out in a BD Falcon® 5 layer multilayer flask (Corning), and transfected with a total of 250 µg plasmid vectors (125 + 125 µg for A/B and Bn/Bs vaccine proteins, and 250 for C_H3 vaccine proteins) using 500 µg Polyethylenimine. Vaccine proteins were purified from HEK293E supernatant using a column with DNP (dinitrophenyl)-lysine-Sepharose (Sigma). The column was washed with PBS and 0,05 M hapten analogue N-carbobenzoxy (CBZ)-glycine (pH 7.4, Merck) in 0.5 M Saline. Vaccine proteins were eluted with 0.05 M DNP-glycine (pH 7.3, Sigma) in PBS, and run through a Dowex 1x8-100 Cl form ion-exchange resin (Sigma) to remove the DNP hapten. The concentration of purified proteins was verified by ELISA using 1 µg/ml DNP-BSA as coat and detected with 1 µg/ml biotinylated anti-λ mAb (9A8) or anti-

MIP1α mAb (MAB450), and 1 μg/ml anti-A/B (2H11) as coat and detected with 1 μg/ml biotinylated anti-scFv[315] mAb (Ab2-1.4).

DC-enriched bone marrow cells and chemotaxis assays

For preparation of Flt3L-induced DCs, bone marrow cells were harvested by flushing tibiae and femurs with medium. The cell suspension was filtered through a 70 μm Nylon cell strainer and seeded at a concentration of 2×10^6 cells per ml, 5 ml per well in a six-well plate. Then 0.1 μg/ml of Flt3L (Peprotech, NJ) was added and the cells were incubated for 9 d at 37°C 5% CO_2. Semiadherent cells were then harvested and used for chemotaxis. 200 μl medium (RPMI1640 with 1% bovine serum albumin) containing various purified vaccine proteins were added to the bottom wells of Transwell plates (5 μm pore polycarbonate membrane). 200 μl ($2x10^6$) Flt3L-induced DC cells were added to the upper wells and incubated 4 hours at 37°C. Cells that migrated to the bottom wells were analyzed by flow cytometry. The cDC1 population was gated based on staining with anti-CD45R/B220 (RA3-6B2; Tonbo Biosciences), anti-CD11c (N418; Tonbo Biosciences), anti-CD11b (M1/70; Tonbo Biosciences) and anti-CD24 (M1/69; BioLegend). In some of the chemotaxis experiments, 2 μg of either anti-MIP1α mAb (MAB450, R&D Systems) or anti-Xcl1 mAb (LS-C16241, Lifespan Biosciences) were added together with the purified protein (2 nM or 0.05 μg). The chemotactic index was calculated as fold increase in the number of cells migrating in the presence of chemotactic factors by the various vaccine proteins as compared to spontaneous cell migration (medium alone or mock).
