# Supplementary material for: A DNA Vaccine That Encodes an Antigen-Presenting Cell-Specific Heterodimeric Protein Protects against Cancer and Influenza
Source: Mol Ther Methods Clin Dev. 2020 Jan 25;17:378–92. doi: 10.1016/j.omtm.2020.01.007 (PMC7044496; doi:10.1016/j.omtm.2020.01.007)
Supplement: Document S2. Article plus Supplemental Information [file mmc2.pdf]

Molecular Therapy
Methods & Clinical Development
Original Article

A DNA Vaccine That Encodes an Antigen-Presenting Cell-Specific Heterodimeric Protein Protects against Cancer and Influenza

Ranveig Braathen,[1,2] Heidi Cecilie Larsen Spång,[1,2] Daniëla Maria Hinke,[1] Jana Blazevski,[1] Sonja Bobic,[1] Even Fossum,[1] and Bjarne Bogen[1]

[1]K.G. Jebsen Centre for Influenza Vaccines Research, Institute of Clinical Medicine, University of Oslo and Oslo University Hospital, 0027 Oslo, Norway

Immunogenicity of DNA vaccines can be increased by constructing the DNA in such a way that it encodes secreted homodimeric fusion proteins that target antigen-presenting cells (APCs). In this study, we have developed novel APC-targeting vaccine molecules with an increased flexibility due to introduction of a heterodimerization motif. The heterodimeric proteins permit four different fusions within a single molecule, thus allowing expression of two different APC-targeting moieties and two different antigens. Two types of heterodimeric fusion proteins were developed that employed either the ACID/BASE or the Barnase/Barstar motifs, respectively. The ACID/BASE heterodimeric vaccines conferred protection against challenges with either influenza virus or tumor cells in separate preclinical models. The ACID/BASE motif was flexible since a large number of different targeting moieties and antigens could be introduced with maintenance of specificity, antigenicity, and secretion. APC-targeting ACID/BASE vaccines expressing two different antigens induced antibody and T cell responses against either of the two antigens. Heterodimeric ACID/BASE DNA vaccines were of approximately the same potency as previously reported homodimeric DNA vaccines. The flexibility and potency of the ACID/BASE format suggest that it could be a useful platform for DNA vaccines that encode APC-targeting fusion proteins.

INTRODUCTION

Naked DNA vaccines are attractive due to their ease of construction, cost-effective manufacture, and safety profile. Several veterinary DNA vaccines are currently licensed, e.g., for West Nile virus in horses,[1] melanoma in dogs,[2] and infectious hematopoietic necrosis virus in salmon.[3] DNA vaccines against papilloma virus have been tested with promising results in women with cervical intraepithelial neoplasia,[4] but there are as of yet no licensed DNA vaccines for humans.[5] Despite these encouraging results, DNA vaccines suffer from low immunogenicity. In particular, it has been difficult to translate promising results in rodents to larger animals and humans.[6] Delivery with electroporation (EP)[7] or noninvasive needle-free jet delivery systems[8] partially overcomes the low immunogenicity of naked DNA.

Immune responses to DNA vaccines are due to the protein antigen that the DNA encodes. A way to increase immunogenicity of protein antigens is to target them against antigen-presenting cells (APCs) by use of antibody-antigen conjugates[9–11] or antibody-antigen fusion proteins.[12,13] In a logical extension, it was later shown that DNA constructs encoding a secreted APC-specific fusion protein resulted in increased T cell and antibody responses.[14–19] Moreover, the specificity of the DNA-encoded fusion protein for APCs influenced the type of immune responses that were induced.[20–23] For example, whereas anti-major histocompatibility complex class II (MHCII) and anti-CD11c targeting units induced responses skewed against T helper 2 (Th2) cells/immunoglobulin G1 (IgG1), MIP1α (CCL3) and Xcl1 induced predominantly Th1/CD8/IgG2a responses.[20,22,23] In further experiments, it was shown that inhibiting endocytosis of the APC-targeting vaccine protein resulted in increased antibody responses, presumably because an APC-B cell synapse of longer duration supports a stronger B cell response.[24] These results suggest that by careful selection of targeting moieties in the APC-targeting DNA vaccines, it should be possible to tailor vaccines in such a way that they preferentially induce immune responses that match those required to eliminate a certain pathogen.

In our previous studies, we used DNA vaccines that encode bivalent vaccine proteins (Vaccibodies) where each chain in the homodimer contains a targeting unit connected to the antigenic unit via a homodimerization unit composed of a shortened Ig hinge linked to a C_H3 domain. Given that the identity of the targeting moiety influences immune responses,[20,22,23] and the desirability of expressing several antigens in the same molecule, we decided to generate novel DNA vaccines that encode heterodimeric vaccine proteins with four arms available for fusions, e.g., two for different targeting moieties and

Received 13 January 2020; accepted 13 January 2020;
https://doi.org/10.1016/j.omtm.2020.01.007.

[2]These authors contributed equally to this work.

Correspondence: Ranveig Braathen, K.G. Jebsen Centre for Influenza Vaccine Research, Institute of Clinical Medicine, University of Oslo and Oslo University Hospital, 0027 Oslo, Norway.
E-mail: ranveig.braathen@medisin.uio.no
Correspondence: Bjarne Bogen, K.G. Jebsen Centre for Influenza Vaccine Research, Institute of Clinical Medicine, University of Oslo and Oslo University Hospital, 0027 Oslo, Norway.
E-mail: bjarne.bogen@medisin.uio.no

 © 2020 The Author(s).
This is an open access article under the CC BY license (http://creativecommons.org/licenses/by/4.0/).

two for different antigens. Two heterodimerization motifs were explored: (1) the ACID/BASE (A/B)-modified leucine zipper motif[25] and (ii) the Barnase/Barstar (Bn/Bs) bacterial motif.[26] We found that the A/B-based DNA vaccines were superior to Bn/Bs-based vaccines and at least on par with the C_H3-based homodimeric vaccine molecules. Moreover, the A/B-based format appeared to be flexible since it readily accepted introduction of different targeting moieties and antigens.

RESULTS

An APC-Targeting DNA Vaccine Based on the A/B Heterodimerization Motif

We constructed a novel type of an APC-targeting DNA vaccine that at the protein level uses a Jun/Fos-based leucine zipper with modified α helices enriched for either acidic or basic amino acids to facilitate non-covalent heterodimerization[25] (Figure 1A). The ACID (A) and BASE (B) chains of this motif were each linked to the human IgG3 (hIgG3) hinge 1 region that contains two cysteines for generation of covalent disulfide bonds. APC-specific targeting units were linked amino terminally to the hinge of the A and B chains, respectively. The antigenic units were linked to the carboxyl-terminal end of either of the two chains (Figure 1A). The two plasmids that together encode the A/B vaccine protein are depicted to the right of the protein structure (Figure 1A). For the purpose of comparison we also produced DNA vaccines based on the previously published bacterial Bn/Bs heterodimerization motif[26] and the Ig C_H3 homodimerization motif[17] (Figure 1A).

Construction of an A/B Heterodimeric DNA Cancer Vaccine

To test efficacy of the different formats as cancer vaccines, the three types of vaccine molecules were equipped with MIP1α as a targeting unit (Figure 1A). MIP1α binds CCR1, CCR3, and CCR5 chemokine receptors expressed on APCs and has previously been shown to induce strong T cell responses when used in the C_H3-based vaccine format.[16,20] The tumor-specific antigen expressed in the antigenic unit was single-chain variable fragment $(scFv)^{315}$, which corresponds to the variable (V) regions of myeloma protein M315 produced by the MOPC315 multiple myeloma tumor, linked in a scFv format.[16,17,27,28] In the non-targeting control vaccines, MIP1α was replaced with $scFv^{\alpha NIP}$ specific for the hapten NIP (4-hydroxy-3-iodo-5-nitrophenylacetic acid) that should not be present in the body (Figure 1A).

To ensure that the three formats of DNA vaccines were efficiently translated into vaccine protein, proper combinations of plasmids were transiently transfected into human embryonic kidney 293E (HEK293E) cells. Supernatants (SNs) were analyzed by ELISAs and western blots for the presence of vaccine proteins. For all three vaccine formats, plasmids were translated into vaccine proteins that were recognized by conformation-specific monoclonal antibodies (mAbs) or haptens with an expected pattern (Figures S1A and S1B). Vaccine proteins had an expected size (Figure S1C). All three MIP1α vaccine proteins attracted the $CCR1^+CCR5^+$ cell line Esb-MP (Figure S1D) but also Flt3L induced dendritic cell (DC)-enriched bone marrow cells from BALB/c mice (Figure S1E). Both vaccine proteins either in supernatants or as affinity-purified proteins had chemotactic activity (Figures S1D and S1E). The chemotactic activity of MIP1α was similar for the A/B heterodimers and the C_H3-based vaccine, whereas the Bn/Bs heterodimer had weaker activity. Collectively, these data suggest that the moieties in the targeting and antigenic units folded correctly as fusions with the three dimerization units, including the novel A/B heterodimeric unit.

The A/B Heterodimeric DNA Vaccine Induces Protection against Subcutaneously Injected Multiple Myeloma Cells

The three types of targeting DNA vaccines, all expressing the tumor-specific antigen $scFv^{315}$, were then tested for their ability to induce protection against MOPC315 myeloma cells. BALB/c mice were DNA vaccinated/EP once and challenged 14 days later with MOPC315 cells injected subcutaneously (s.c.). Survival curves for the various groups of vaccinated mice (Figure 1B), as well as the levels of the tumor-specific myeloma protein M315 in sera (Figure 1C), demonstrate that vaccination with the MIP1α-targeting A/B heterodimer conferred a significant (60%) survival while all mice receiving non-targeting control vaccines died. Results with the C_H3-based homodimer were similar while the Bn/Bs heterodimer gave no protection at all (Figures 1B and 1C). These results show that the A/B heterodimeric DNA vaccine confers equal protection against a tumor challenge compared with the previously published C_H3-based homodimer.[16] Moreover, the MIP1α targeting moiety is crucial for the efficacy of the vaccine. Due to its ineffectiveness in this tumor model, Bn/Bs-based DNA vaccines were excluded from further experiments.

The A/B Heterodimeric DNA Cancer Vaccine Induces Protection against Intravenously Injected Multiple Myeloma Cells

Human multiple myeloma is characterized by expansion of malignant plasma cells in the bone marrow. We have generated a mouse model for multiple myeloma where MOPC315.BM cells injected intravenously (i.v.) induces bone disease.[29] Mice vaccinated once with DNA vaccines were challenged i.v. with the MOPC315.BM variant 14 days later. The MIP1α-targeting A/B heterodimeric vaccine gave significantly better survival (∼70%) than did the non-targeting control (∼20%) and an antigen control that expressed a scFv from an irrelevant B cell lymphoma (0%). The MIP1α-A/B heterodimeric vaccine gave slightly better survival than did the homodimeric C_H3-based equivalent (∼50%), although the difference was not significant (Figure 2A). The levels of myeloma protein in sera, as a readout for tumor load, reflected the survival curves (Figure 2B). In summary, the heterodimeric A/B DNA vaccine conferred protection against an i.v. tumor challenge, and the protection depended on APC targeting by MIP1α.

The Tumor-Protective Effect of the MIP1α-A/B Heterodimeric DNA Vaccine Is Due to Induction of T Cells

To investigate the contribution of T cells to the vaccine-induced protection, we used the MOPC315.BM variant stable transfected with firefly luciferase (Luc), which permits easy tracking of tumor load with noninvasive imaging.[29] In a first experiment, mice vaccinated once with a MIP1α-C_H3-$scFv^{315}$ homodimeric vaccine were significantly protected compared to mice immunized with the antigen control

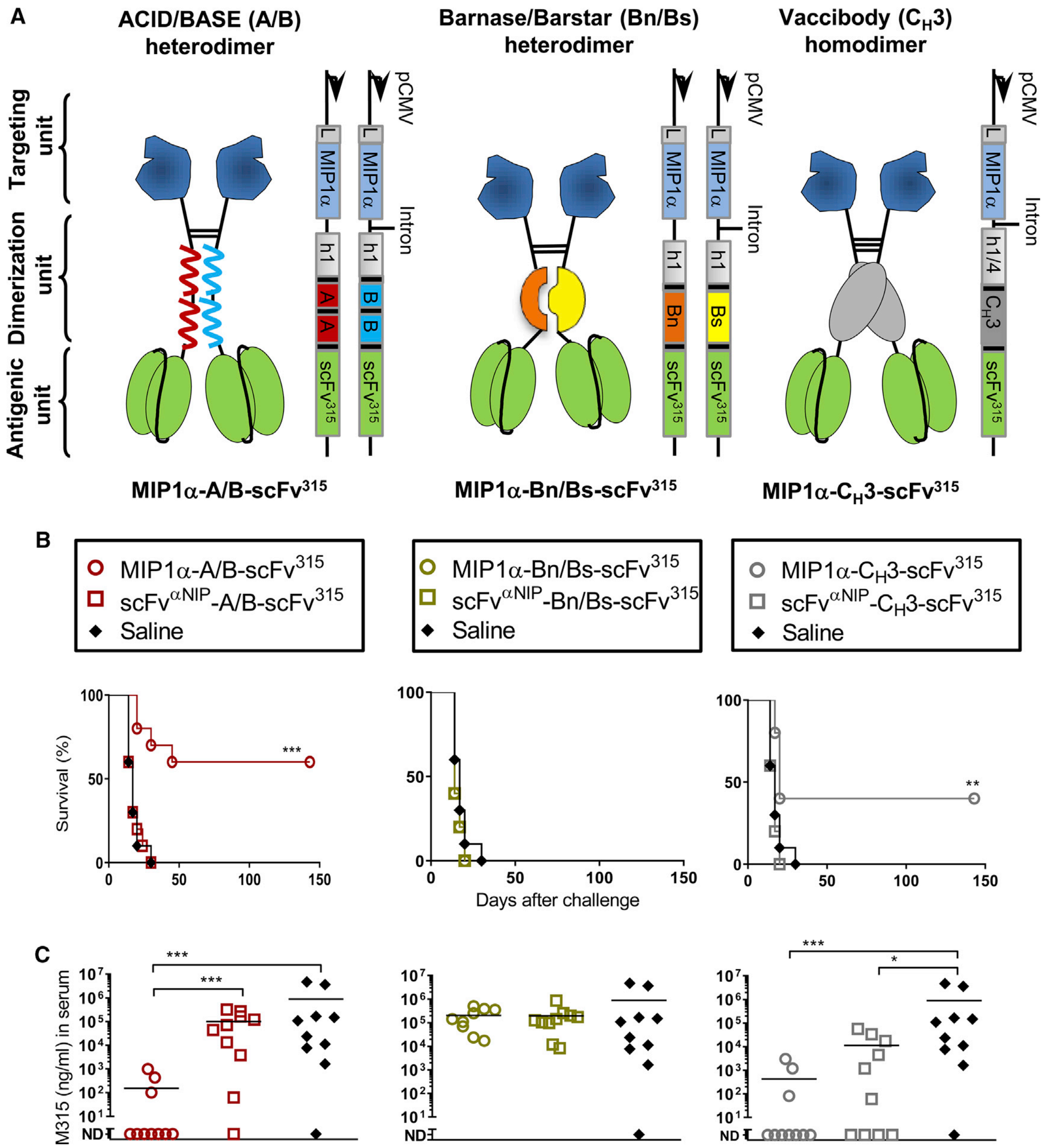

Figure 1. A MIP1α-Targeting ACID/BASE Heterodimeric DNA Vaccine Induces Protection against a s.c. Tumor Challenge

(A) Targeting vaccine molecules are compared. The vaccine molecules are formed by two chains consisting of a targeting unit (blue), a dimerization unit (different colors), and an antigenic unit (green). The dimerization unit differs in the three formats. The ACID/BASE (A/B) heterodimer (left) uses the human (h)1 hinge region hγ3 exons fused to a duplicated Jun/Fos-based leucine zipper with modified α helices containing either increased amounts of acidic (A, red) or basic (B, blue) amino acids. The Barnase/Barstar (Bn/Bs) heterodimer (middle) uses the h1 hinge region hγ3 exons fused to either Bn (orange) or Bs (yellow). The homodimeric vaccine (right) uses the h1 and h4 hinge region fused to C_H3 hγ3 (gray, to the right). The targeting unit is in all three cases the chemokine MIP1α while the antigenic unit is a tumor antigen, the variable (V) regions of mouse myeloma protein M315 assembled in a $scFv^{315}$ format. The theoretical disulfide bonds in the hinge regions are indicated. The DNA plasmids encoding the two chains of the vaccine proteins are indicated on the right side of the vaccine molecules. The plasmids contain a CMV promoter (pCMV) and a leader (L, gray box). Short linkers (black boxes) increase the flexibility between the various units in the protein. (B) Mice were vaccinated once with 100 μg (A/B and Bn/Bs heterodimer, two plasmids) or 50 μg (C_H3-based homodimer, one plasmid) of DNA i.m. combined with electroporation (EP). After 14 days, mice were challenged with 10^5 MOPC315 tumor cells s.c. Survival curves of vaccinated mice (n = 10/group; $^{**}p < 0.01$, $^{***}p < 0.001$, Mantel-Cox analysis, targeting group compared with non-targeting group). (C) Levels of tumor-specific myeloma protein M315 in sera on day 14 after challenge ($^{*}p < 0.05$, $^{***}p < 0.001$, Mann-Whitney test, two-tailed; ND, not detectable).

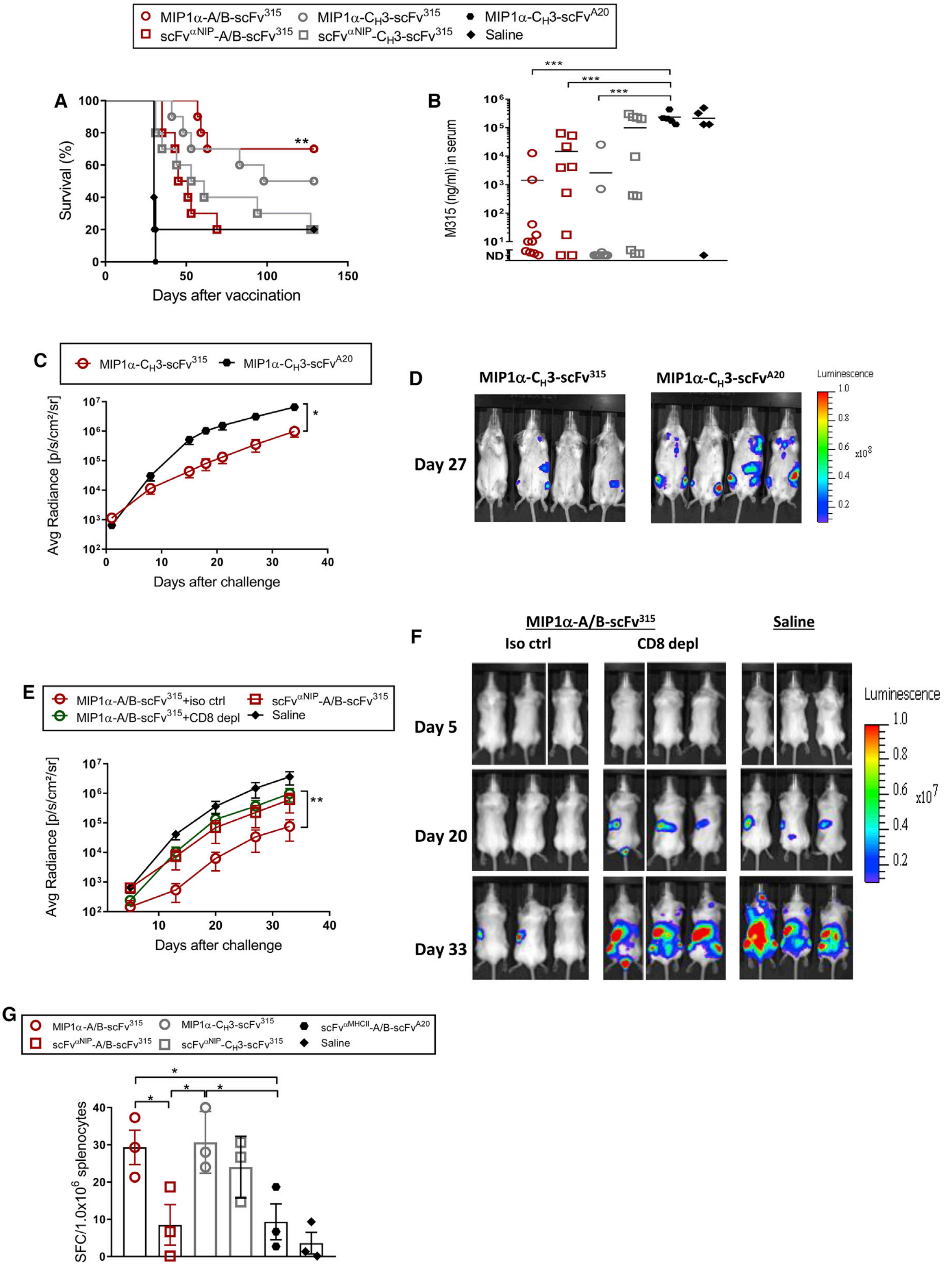

(legend on next page)

(scFv[A20], Figures 2C and 2D). In a second experiment, mice vaccinated once with the MIP1α-A/B-scFv[315] heterodimer were significantly protected compared to the non-targeted control (Figures 2E and 2F). Although performed in two different experiments, the protection conferred by C_H3-based homodimers and A/B-based heterodimers appears similar, consistent with the direct comparisons in Figures 1 and 2A. To test the contribution of $CD8^+$ T cells, mice immunized with the heterodimeric format were from day 12 injected every second day with either depleting anti-CD8 mAbs or isotype-matched control mAbs (for efficiency of depletion, Figure S2). The protection elicited by immunization with the A/B heterodimer was reversed by depletion of $CD8^+$ T cells that most likely recognize an idiotope (Id) sequence contained in scFv[315] (Figures 2E and 2F). Injection of immunized mice with anti-CD4 mAbs caused a reduced growth of MOPC315.BM.LUC cells, most likely due to an unexpected CD4 expression on MOPC315 cells (data not shown). Thus, we cannot say whether also $CD4^+$ T cells contributed to tumor protection elicited by A/B-based immunization.

By use of T cell receptor (TCR)-transgenic mice with $CD4^+$ T cells specific for residues 91–101 of the V_L sequence of scFv[315], it has previously been shown that Id-specific $CD4^+$ T cells can eradicate MOPC315 cells.[28,30] $CD4^+$ T cells of this Id specificity are highly infrequent in normal mice.[31] Therefore, we reconstituted BALB/c mice with TCR-transgenic T cells prior to immunization, as previously described.[15] 11 days after a single DNA vaccination of reconstituted mice, splenic Id-specific $CD4^+$ T cells reactive to the Id peptide/MHCII were detected in an interferon (IFN)-γ T cell enzyme-linked immunospot (ELISPOT) assay. Immunization with either MIP1α-A/B-scFv[315] or MIP1α-C_H3-scFv[315] vaccines elicited significantly increased IFN-γ responses of similar magnitudes, suggesting an equal ability to stimulate $CD4^+$ T cells (Figure 2G).

Protection against Influenza Challenge by the Novel Heterodimeric Vaccine

We next tested if the novel A/B heterodimeric DNA vaccine could induce protection against influenza virus. Hemagglutinin (HA) from the strain PR8 (A/PR/8/34 (H1N1)) was used as antigen. The MIP1α targeting unit was exchanged with a targeting unit that binds the MHCII molecule I-E^d in BALB/c mice; this targeting unit is called scFv[αMHCII]. The reasons for these choices are that HA is a major antigen on influenza virus, that antibodies against HA are important for protection, and that previous experiments with homodimeric C_H3-based DNA vaccines have shown that MHCII targeting results in high levels of antibodies.[20,22,32] As a non-targeting control, we used identical molecules but with scFv[αNIP] in the targeting unit.

BALB/c mice were DNA vaccinated/EP once. The levels of HA-specific IgG1 and IgG2a were measured in sera 6 weeks later. The MHCII-targeting vaccine induced significantly higher levels of HA-specific IgG1 and IgG2a antibodies compared to the non-targeting control (Figures 3A and 3B).

After another 9 weeks, vaccinated mice were challenged intranasally with a lethal dose of PR8 virus. A weight loss of 20% was defined as the humane endpoint at which time point the mice were euthanized. The results show that the mice vaccinated with the MHCII-targeting vaccine did not lose weight and stayed healthy, whereas the mice vaccinated with the non-targeting control got sick, lost weight, and had to be euthanized (Figures 3C and 3D). These findings demonstrate that MHCII targeting of the A/B HA heterodimer increases the levels of anti-HA antibodies and protection against virus, compared to the non-targeting control.

We next directly compared the A/B HA heterodimer and the C_H3-based homodimer in the PR8 influenza model. Both vaccines induced similar amounts of HA-specific IgG1 and IgG2a measured in sera 4 weeks after immunization (Figures 3E and 3F). Mice immunized with either vaccine format were completely protected against a challenge with the PR8 virus (Figures 3G and 3H). These results suggest that the two vaccine formats are equally efficient at inducing HA-specific antibodies and protection against influenza virus.

The A/B Heterodimeric DNA Vaccine Is a Flexible Vaccine Platform

The A/B heterodimerization unit forces two different chains to combine into a bivalent protein vaccine linked by covalent and

Figure 2. A MIP1α-Targeting A/B Heterodimeric DNA Vaccine Induces T Cell Responses and Protection against an i.v. Challenge with Bone Marrow-Homing MOPC315.BM Multiple Myeloma Cells

(A) Mice were vaccinated with 100 μg (A/B heterodimer, two plasmids) or 50 μg (C_H3-based homodimer, one plasmid) of DNA i.m./EP. After 14 days, mice were challenged i.v. with 10^4 MOPC315.BM cells. Survival curves of the vaccinated mice (n = 5–10/group; **$p < 0.01$, Mantel-Cox analysis, targeting compared with non-targeting groups). (B) Blood samples were harvested on day 36 after challenge and analyzed for the myeloma protein M315 in ELISA (mean; ***$p < 0.001$, Mann-Whitney test, two-tailed; ND, not detectable). (C and D) Mice were immunized i.m. with 50 μg of DNA/EP. 2 × 105 MOPC315.BM.Luc tumor cells were injected i.v. 14 days later. (C) Luminescence emitted from tumor cells (mean of dorsal and ventral side) as a function of time (n = 7 for MIP1α-C_H3-scFv[315] and n = 4 for MIP1α-C_H3-scFv[A20], mean ± SEM; **$p < 0.01$, two-way ANOVA). (D) Ventral bioluminescent signals in representative mice on day 27 after challenge. (E and F) Mice were immunized i.m. with 100 μg (two plasmids) of DNA/EP. Starting from day 12, mice were injected every other day for 14 days and after that once a week with 100 μg of depleting mAb against CD8 or isotype control (iso ctrl) mAbs. 2×10^5 MOPC315.BM.Luc tumor cells were injected i.v. on day 14 after vaccination. (E) Luminescence emitted from tumor growth sites (mean of dorsal and ventral sides) as a function of time (n = 6 for MIP1α-A/B-scFv[315] iso ctrl and scFv[αNIP]-A/B-scFv[315]; n = 8 for MIP1α-A/B-scFv[315] CD8 depleted; n = 4 for NaCl, mean ± SEM; **$p < 0.01$, two-way ANOVA for comparison of CD8-depleted and iso ctrl groups at day 33). (F) Dorsal bioluminescent signals in representative mice in the MIP1α-A/B-scFv[315] vaccinated group of either the iso ctrl or CD8-depleted group, and saline group (n = 3), taken on days 5, 20, and 33 after challenge. (G) M315 L-chain (λ2[315])-specific TCR-transgenic $CD4^+$ T cells (3×10^6) were transferred i.v. to BALB/c mice, followed the next day by the indicated vaccinations (i.m.). After 11 days, spleens were harvested and analyzed for IFN-γ-secreting T cells (ELISPOT) after stimulation with a MHCII-restricted λ2[315] CDR3 peptide (ALWFRNHFVFGGGTKVT, amino acids 91–107). The spleens were pooled within the groups (n = 4/group) and run in technical triplicate (mean ± SEM; *$p < 0.05$, Mann-Whitney test, one-tailed).

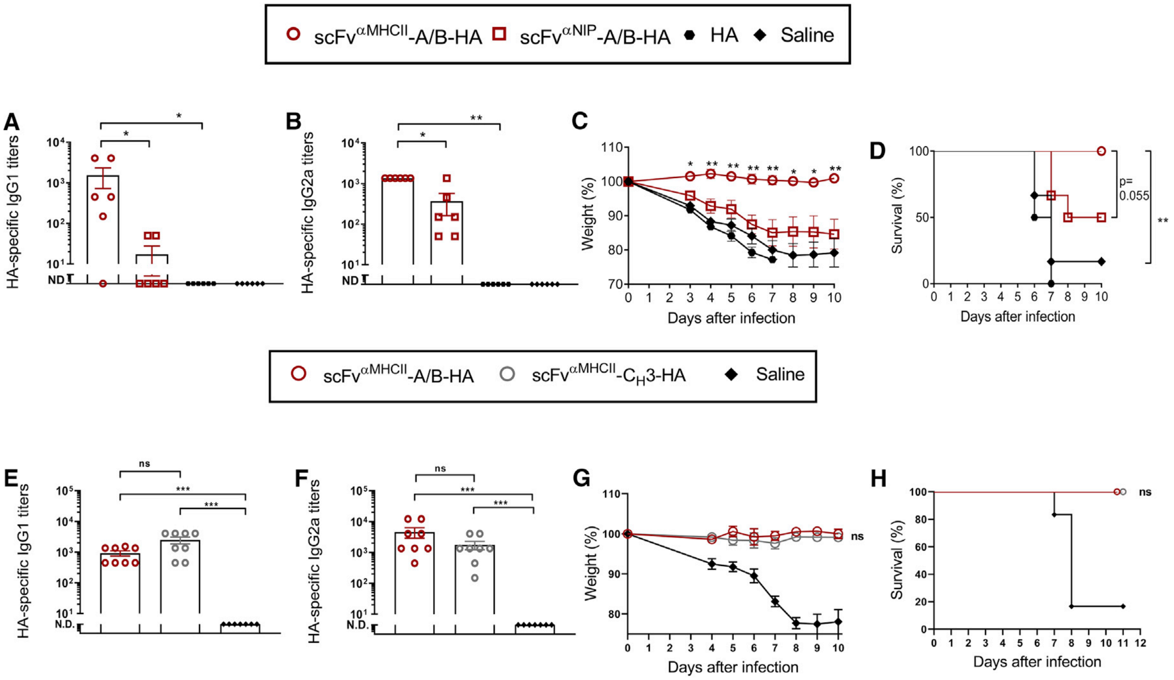

Figure 3. MHCII-Targeting A/B Heterodimeric and C_H3 Homodimeric DNA Vaccines Both Confer Complete Protection against Influenza Infection

In (A)–(D), BALB/c mice were vaccinated with 100 μg of (two plasmids) DNA i.m./EP with the indicated A/B heterodimeric vaccines expressing HA from PR8 (H1N1). In (E) and (F), A/B heterodimeric and C_H3-based homodimeric vaccines were compared, using 100 μg (A/B heterodimer, two plasmids) and 50 μg (C_H3 homodimer, one plasmid) of DNA i.m./EP. (A, B, E, and F) Levels of HA-specific IgG1 (A and E) and IgG2a (B and F) serum antibodies 6 weeks after immunization (mean ± SEM, **p < 0.01, Mann-Whitney test; ND, not detectable). (C and G) 15 (C) or 4 (G) weeks after vaccination, the mice were infected with a lethal dose of PR8 influenza virus (7.5 × LD_{50}). Weight was followed for 10 days (mean ± SEM; *p < 0.05, **p < 0.01, Mann-Whitney test, comparing targeting to non-targeting vaccines). (D and H) Survival curves of the vaccinated mice. Non-significant (ns, p = 0.0549) or significant p values are indicated (**p < 0.01, Mantel-Cox test). n = 6/group in (A)–(D), n = 8/group in (E)–(H). Weight loss of 20% was defined as the humane endpoint, at which time point the mice were euthanized.

non-covalent forces. Thus, four different fusions expressed in a single molecule are theoretically possible. In the present work, we have used the amino terminal fusions for targeting purposes and the carboxyl-terminal fusions for antigens. Thus, each A/B heterodimeric molecule may have either two identical or two different targeting units, and either two identical or two different antigenic units. A premise for the above is that all the four arms are suitable for fusions. To test this, we co-transfected HEK293E cells with combinations of plasmids encoding A and B chains with a variety of different targeting units and antigens (Figure 4). Supernatants were tested in a panel of sandwich ELISAs where the coat and detection reagents should bind to the various parts of the A/B vaccine molecule, either in *cis* or in *trans*. A positive signal in the sandwich ELISAs would indicate that the two tested moieties, detected respectively by the coat and the detection reagent, are linked in the vaccine molecule.

Four experiments were performed (Figures 4A–4D). The experiments employed four different targeting units (in addition to those mentioned above, a chemokine Xcl1 previously used for targeting Xcr1$^+$ DCs[23] was included). Five different antigenic units were used (in addition to those mentioned above, novel antigens were ovalbumin [OVA], the fluorescent protein mCherry, and HA of Cal07 [A/California/07/09 (H1N1)] influenza virus). In some experiments, vaccine molecules with a C_H3-based homodimerization motif were included. A number of conclusions can be made from the sandwich ELISAs: (1) a signal was always obtained according to predictions; (2) a signal was never obtained when not expected; (3) all four arms appeared suitable for fusions with maintenance of secretion and expression of conformational determinants; and (4) the two chains assembled in heterodimers since a *trans* signal was always obtained according to predictions. It may be concluded that the A/B vaccine format is robust in the sense that a number of different targeting units and antigenic units may be introduced with maintenance of secretion, conformation, and heterodimeric structure. The largest antigen we have inserted in the A/B heterodimer with maintenance of secretion and immunogenicity is 523 aa (HA). Thus, the A/B format is flexible and could be useful for constructing vaccines for a number of different antigens relevant to infectious diseases and cancers.

The respectively acidic and basic charges of the A or B dimerization motif suggests that the two chains composing an A/B heterodimer preferentially pair through an electrostatic interaction.[25] To clarify whether monomers could be produced as well, we performed transient transfection of HEK293E cells with either (1) A and B plasmids that together encode A/B heterodimeric vaccine protein or (2) with only one of the A or B plasmids. PR8 HA was used as antigen while

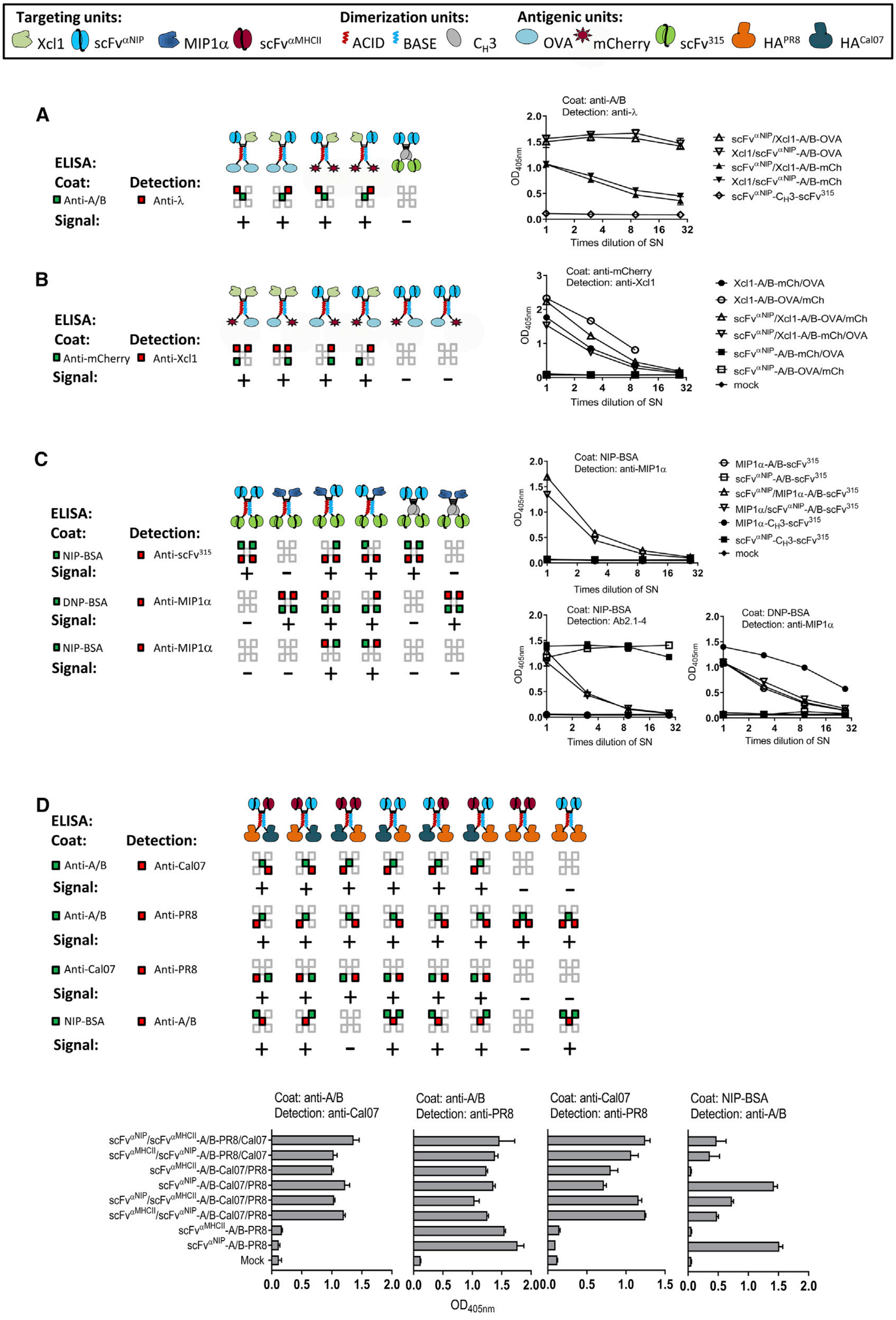

(legend on next page)

Cal07 HA served as a specificity control. Upon transfection with only one plasmid, both the A and B plasmids were translated into monomeric vaccine proteins detected in ELISAs (Figure S3A, left). These monomers, and especially chains with the B motif, were able to form homodimers as suggested by a western analysis (Figure S3B). Although this experiment demonstrates that monomers can be secreted *in vitro*, and that B chains can dimerize, this result was obtained in the absence of competition by an A chain. Under conditions of competition, where the B chain can pair with either an A chain or a B chain, the A chain was preferred (Figure S3A). Thus, it is likely that when immunizing with A and B plasmids, A/B heterodimeric vaccine protein is preferentially formed.

A Single Targeting Unit per A/B Heterodimeric Vaccine Molecule Is Sufficient for Chemotaxis and Induction of Antigen-Specific IgG1 and IgG2a Responses

We proceeded to test the importance of one versus two targeting units in a single heterodimeric A/B vaccine molecule. First, we tested whether A/B vaccine proteins with either none, one, or two MIP1α targeting units could induce chemotaxis *in vitro*. A single targeting unit per vaccine molecule was sufficient to induce chemotaxis irrespective of whether the MIP1α-targeting unit was attached to the A or the B arm (Figure 5A). Similarly, one MIP1α moiety in purified A/B vaccine proteins was sufficient for chemotaxis of DC-enriched bone marrow cells from BALB/c mice (Figure S4A). The MIP1α-containing A/B heterodimers attracted conventional DCs of type 1 (cCD1, Figure S4A), consistent with a previous publication.[33] To demonstrate that a single A/B molecule can carry two distinct chemokines that each have chemotactic activity, we employed the Xcl1 chemokine known to induce chemotaxis of Xcr1^{+} conventional type 1 DC (cDC1) cells.[33] MIP1α/Xcl1-containing A/B vaccine protein attracted cDC1 cells, and neither an excess of anti-MIP1α nor anti-Xcl1 mAbs inhibited chemotaxis. This result is consistent with both MIP1α and Xcl1 chemokines being functional within a single A/B vaccine molecule (Figure S4B). Next, we tested whether A/B DNA vaccines encoding vaccine protein with either one or two MIP1α-targeting units differed in their ability to induce antigen-specific IgG1 and IgG2a responses against the scFv^{315} multiple myeloma antigen that was bivalently expressed (on both A and B arms). No difference was found in serum antibody levels in immunized mice (Figure 5B). Similar results were found when testing either one or two MHCII-targeting units in molecules bivalent for scFv^{315} (Figure 5C). These results suggest that a single targeting unit is sufficient for the DNA vaccines to elicit antibodies.

Single Heterodimeric A/B Molecules That Express Two Different Antigens Elicit Antibody Responses against Either

We next tested whether two different antigens expressed in a single A/B heterodimeric molecule are both able to elicit antibodies. HA from PR8 and Cal07 influenza viruses was fused to respectively the A and B arms (or vice versa). These two H1 HAs do not elicit cross-reactive antibodies. The MHCII-specific targeting unit was bivalently expressed, i.e., on both the A and B arms. In immunized BALB/c mice, levels of anti-HA^{PR8} and anti-HA^{Cal07} antibodies were similar, irrespective of whether HA^{PR8} and HA^{Cal07} were attached to the A or B chain. This result indicates that antigens fused to either the A and the B arm elicit antibodies and to a similar extent (Figure 6A).

Single Heterodimeric A/B Molecules That Express Two Different Antigens Elicit T Cell Responses against Either

Finally, we expressed two different antigens, HA from PR8 virus and OVA, in a single A/B heterodimeric molecule, fused to respectively the A and B arms (or vice versa). The vaccine molecule was bivalent for the MHCII-specific targeting unit. Splenocytes of DNA-immunized mice were stimulated *in vitro* with defined MHC class I (MHCI)- or MHCII-restricted synthetic peptides, or complete proteins, of either the HA^{PR8} or OVA origin. IFN-γ-producing cells were detected by ELISPOT. Responses to HA^{PR8} and OVA proteins, or synthetic peptides derived from them, were similar, irrespective of the arm to which the immunizing antigen had been fused (Figure 6B). This result shows that a single heterodimeric vaccine molecule can induce T cell responses toward two separate antigens expressed on the A and B chain, respectively. As for antibody responses, T cell responses were elicited regardless of whether the antigen was fused to the A or B arm.

Two Different Influenza HA Antigens within a Single Heterodimeric A/B Molecule Can Induce Protective Immunity against Either of the Two Corresponding Viruses

Two ways of immunization were compared: (1) co-injection of A and B plasmids encoding an A/B heterodimer where HA^{PR8} was expressed on the A arm while HA^{Cal07} was expressed on the B arm (the heterodimeric molecule bivalently expressed an anti-MHCII targeting unit), and (2) co-injection of two different plasmids encoding anti-MHCII-C_H3 chains that expressed either HA^{PR8} or HA^{Cal07}, respectively. Theoretically, C_H3-based homodimerization in the endoplasmic reticulum (ER) of transfected cells should give three types of molecules that express HA^{PR8}/HA^{PR8}, HA^{PR8}/HA^{Cal07}, and HA^{Cal07}/HA^{Cal07} in a 1:2:1 ratio.[34] The immunized mice were boosted after 5 weeks and PR8- and Cal07-specific IgGs were measured 2 weeks after the boost. The

Figure 4. Diverse Targeting Units and Antigenic Units Are Efficiently Expressed on Both the A and B Arm of the Heterodimeric Protein Vaccine *In Vitro*

(A–D) Targeting units, dimerization units, and antigenic units are indicated by symbols (given in boxes). HEK293E cells were transiently transfected with combinations of various DNA vaccine plasmids, theoretically yielding the vaccine molecules depicted in the top rows. Supernatants were analyzed in sandwich ELISAs as indicated (mean + SD). Expected binding of coat mAb (green) or detection mAb (red) to protein vaccine units (squares) is indicated by colors. Positive or negative signal obtained in sandwich ELISAs are indicated by + or −. (A) The targeting units, Xcl1 chemokine and scFv$^{\alpha NIP}$, were genetically fused to either A or B chains in combination with OVA and mCherry as antigenic units, as indicated. Homodimeric scFv$^{\alpha NIP}$-C_H3-scFv^{315} was included as a negative control. (B) The targeting units, Xcl1 and scFv$^{\alpha NIP}$, were genetically fused to either A or B chains in combination with antigenic units OVA and mCherry, as indicated. (C) MIP1α and scFv$^{\alpha NIP}$ were expressed in all possible combinations in the targeting units of A/B heterodimers and C_H3-based homodimers, as indicated. The antigenic unit, scFv^{315}, was invariable. (D) The targeting units, scFv$^{\alpha MHCII}$ and scFv$^{\alpha NIP}$, were expressed with antigenic units HA from PR8 and Cal07 influenza strains in A/B heterodimers, as indicated.

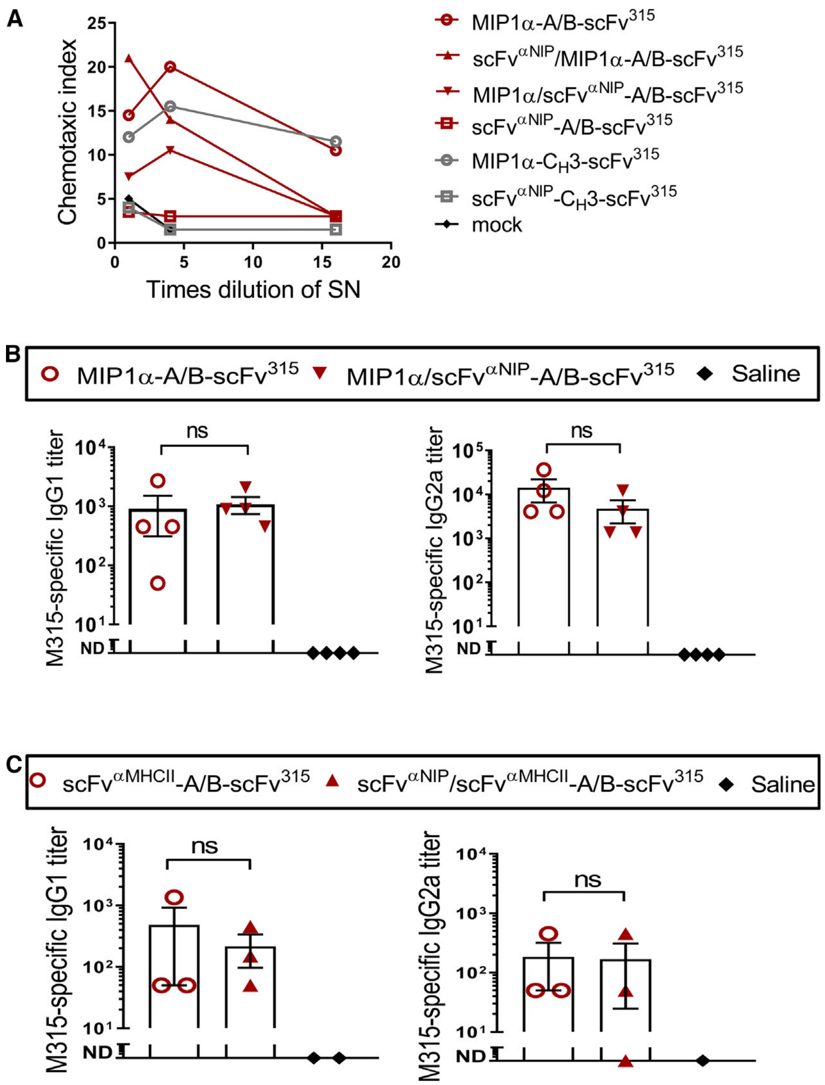

Figure 5. A Single APC-Specific Targeting Unit in the A/B Heterodimeric Vaccine Is Sufficient for Chemotaxis and Induction of Antigen-Specific IgG1 and IgG2a Responses *In Vivo*

(A) Chemotactic activity of the MIP1α targeting unit genetically fused to either the A or B chain of the dimerization motifs, or both, was analyzed *in vitro*. Dilutions of supernatant from transiently transfected HEK293E cells were tested for their ability to attract $CCR1^+$ $CCR5^+$ ESb-MP cells. (B and C) BALB/c mice were vaccinated with 100 μg (two plasmids) of DNA i.m./EP with A/B heterodimeric vaccines that express either one or two MIP1αs (B), or one or two $scFv^{\alpha MHCII}$s (C), as indicated. 36 days (B, n = 4/group) or 14 days (C, n = 3/group, n = 1-2 for saline group) later, antigen (M315)-specific IgG1 and IgG2a were analyzed in sera (mean ± SEM; Mann-Whitney test; ns, not significant; ND, not detectable).

two vaccine formats induced similar amounts of anti-HA^{PR8} and anti-HA^{Cal07} antibodies (Figures 7A and 7B). Moreover, mice immunized with either of the plasmid formulations were completely protected against challenges with either PR8 (Figures 7C and 7D) or Cal07 (Figures 7E and 7F) viruses. Thus, when two different HAs were expressed in a heterodimeric A/B molecule, protective immune responses against both corresponding viruses were observed. Although a similar result was obtained with the mix of two different plasmids encoding C_H3-based homodimers, one cannot conclude that the molecule that expressed HA^{PR8}/HA^{Cal07} conferred immunogenicity and protection since the immunization should also have resulted in formation of HA^{PR8}/HA^{PR8} and HA^{Cal07}/HA^{Cal07} homodimers.

DISCUSSION

APC-targeting heterodimeric DNA vaccines based on the A/B heterodimerization motif appear to be of at least the same potency as previously published DNA vaccines that use an Ig C_H3 homodimerization motif, as tested in a tumor and an influenza model in mice. The four arms available for fusions in the heterodimeric vaccine molecule accept a number of different fusions with maintenance of secretion, conformation, specificity, and antigenicity. Thus, the A/B heterodimeric DNA vaccine format has an increased versatility compared to the C_H3-based homodimer and could thus be useful in a number of applications. Note, however, that not all heterodimerization motifs might work since the Bn/Bs-based vaccine molecules gave comparatively poor responses.

Upon intramuscular (i.m.) DNA vaccination and EP, we have previously shown that transfected muscle cells produce the APC-targeted C_H3-based vaccine protein.[35] After intradermal (i.d.) DNA vaccination and EP, others have found that cells in the *panniculus carnosus* muscle layer in the skin and other cells in the hypodermis are transfected.[36] Subsequent to DNA vaccination, C_H3-based homodimeric vaccine proteins were found in serum of mice but only in the absence of APC targeting. In contrast, when the vaccine molecules could bind MHCII, they were undetectable in serum, presumably due to absorption by APCs.[17] We have performed similar experiments with DNA immunization/EP with the A/B based heterodimers but have not been able to detect vaccine proteins in sera of immunized mice. A reason for this could be that the A/B heterodimeric vaccine proteins are more rapidly degraded than C_H3-based homodimers. If so, this does apparently not preclude immune responses to the A/B-based heterodimers, which were of similar magnitude to that induced by C_H3-based homodimers.

A potential disadvantage of the A/B DNA vaccine is that two separate plasmids need to be injected upon immunization, each of them encoding one of the two different chains (A and B) assembled in the

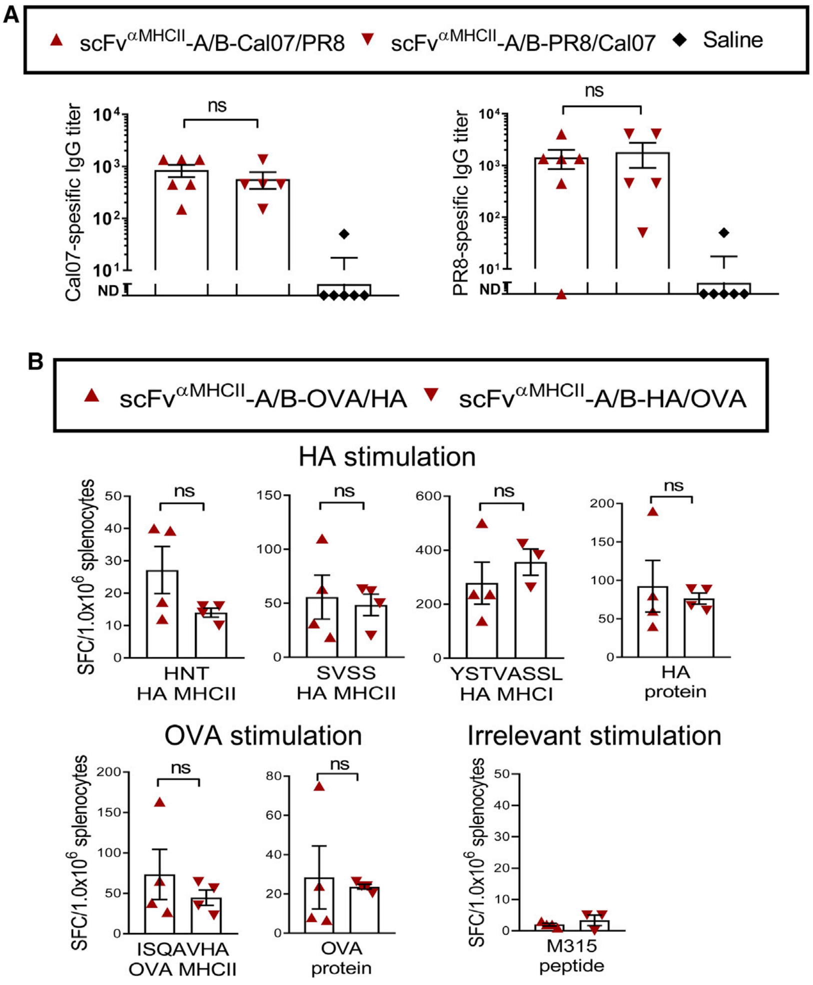

Figure 6. Antigen-Specific Responses Are Induced Irrespective of Whether the Antigen Is Fused to the A or B Arm in the Heterodimeric Vaccine Protein

(A) BALB/c mice were vaccinated with 100 μg (two plasmids) of DNA i.m./EP with A/B heterodimers expressing influenza HA antigen from Cal07 (H1N1) and PR8 (H1N1) connected to either the A or B arm (n = 5-6/group). 14 days after vaccination, the mice were bled and Cal07- and PR8-specific IgGs were analyzed in the sera by ELISA (mean ± SEM; Mann-Whitney test, ns, not significant; ND, not detectable). (B) BALB/c mice were vaccinated with 50 μg (two plasmids) of DNA i.d./EP with A/B heterodimers expressing influenza HA antigen from PR8 (H1N1) and OVA connected to either the A or the B arm (n = 4/group, analyzed as biological replicates). After 11 days, spleens were harvested and analyzed for IFN-γ-secreting T cells (ELISPOT) after stimulation with HA whole protein, MHCII-restricted HA peptides (HNTNGVTAACSHEG or SVSSFERFEIFPK), or MHCI-restricted HA peptide (IYSTVASSL), OVA whole protein, MHCII-restricted OVA peptide (ISQAVHAAHAEINEAGR), or irrelevant peptide ($\lambda 2^{315}$ CDR3 peptide, ALWFRNHFVFGGGTKVT). Mann-Whitney test, two-tailed; ns, not significant.

heterodimeric vaccine protein. The two different plasmids must both be taken up by single cells, resulting in production of the A and the B chains that presumably assemble with non-covalent and disulfide bonds in the endoplasmic reticulum prior to secretion. However, since the A/B heterodimeric vaccine elicits immune responses similar to those of the C_H3-based homodimeric vaccine based on a single plasmid, this might not be a problem. A likely explanation is that a high number of plasmids are injected (1 μg DNA contains 7.5×10^{13} plasmids with a size of 8 kDa), and that a single transfected cell probably takes up many of each of the A and B plasmids with a nearly 50/50 distribution.[37] Consistent with this scenario, joining of two plasmids into a single plasmid that encodes both the A and B chain did not result in improved responses (unpublished data).

The two different chains of the heterodimer, A and B, were found to be expressed *in vitro* when A and B plasmids were transfected separately. Moreover, solitary transfections of the B plasmid resulted in formation of B-B homodimeric proteins under such non-competitive conditions. However, the acidic and basic charge of respectively the A and B motif would suggest that they preferentially bind to each other when both are present.[25] Indeed, peptides having the A and B motif showed 10^5-fold greater propensity for heterodimer formation over homodimeric formation.[38] Consistent with this, we found that A/B heterodimers efficiently formed *in vitro* as analyzed by antibodies binding targeting and antigenic units in *trans*, and by binding of the 2H11 mAb that requires an assembled A/B heterodimer for its binding.[39] Furthermore, we found that heterodimers were preferentially formed under competing conditions where both A and B chains were present.

We have previously shown that the targeting unit can affect the type of immune response that is elicited by the vaccine antigen,[22] most likely reflecting the type of APCs that the targeting unit binds and possibly the particular cell surface molecule that is targeted. Thus, targeting units (Xcl1, MIP1α) that bind to receptors preferentially found on cDC1 cells increases Th1/IgG2a/CD8 responses.[21,22,33] In contrast, targeting units (anti-MHCII, anti-CD11c) that bind to receptors expressed on many DC subtypes, including the cDC2, also increases Th2 and IgG1 responses.[20,22] These observations, made with C_H3-based homodimers, most likely apply to the A/B heterodimers described herein.

It is surprising that having either one or two targeting molecules per A/B vaccine molecule did not have a striking effect on levels of antibody production, T cell responses, or chemotaxis, at least not when employing MIP1α chemokine and anti-MHCII as targeting units. This finding is in line with more limited experiments using heterodimeric vaccine

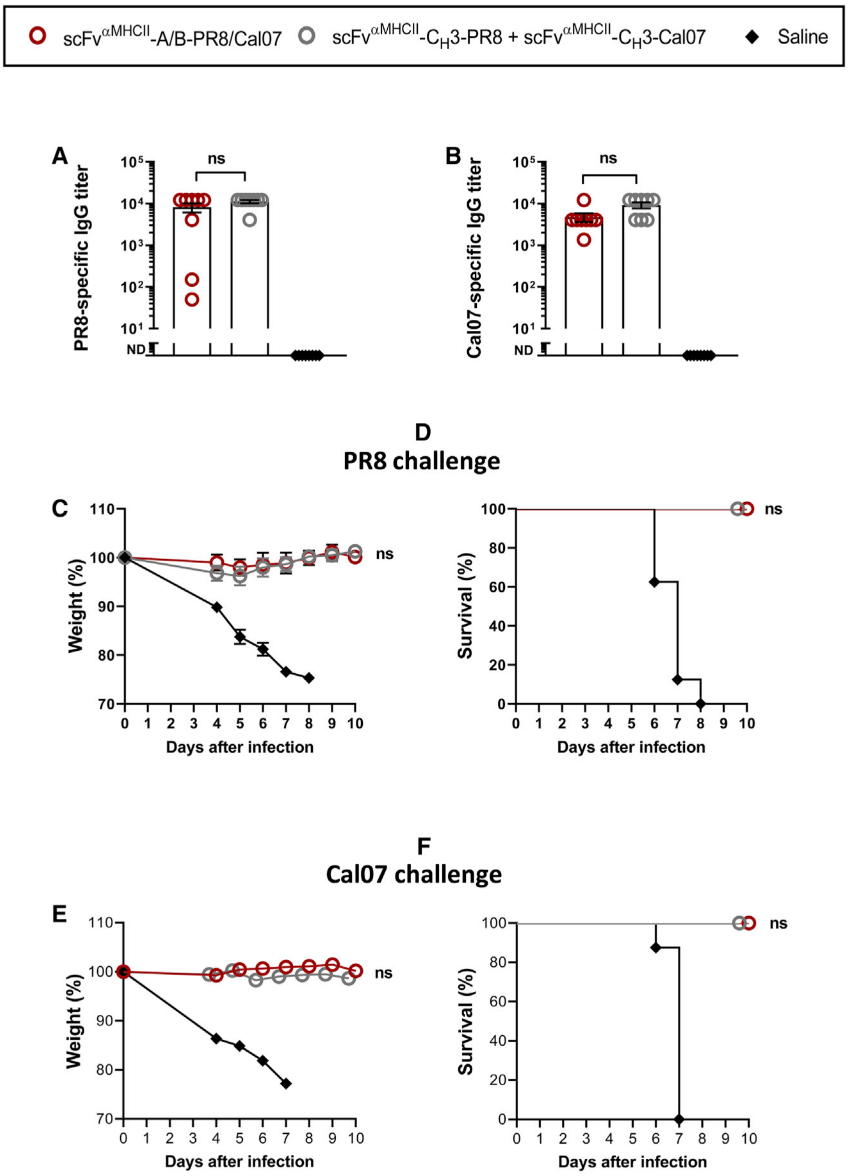

Figure 7. Comparison of A/B Heterodimeric and C_H3-Homodimeric DNA Vaccines Expressing Two Different HAs

BALB/c mice were vaccinated i.m./EP with 100 μg of DNA (two plasmids) encoding either A/B heterodimeric or C_H3 homodimeric vaccine proteins that express HA from both PR8(H1N1) and Cal07(H1N1) as indicated. Mice were boosted after 5 weeks. (A and B) Levels of PR8-specific IgG (A) and Cal07-specific IgG (B) serum antibodies 2 weeks after the boost (mean ± SEM; **p < 0.01 Mann Whitney; ND, not detectable). (C and E) 2 weeks after the boost, the mice were infected with a lethal dose of PR8 (C, 5 × LD_{50}), or Cal07 (E, 5 × LD_{50}) influenza virus. Weight was followed for 10 days. Weight loss of 20% was defined as the humane endpoint, at which time point the mice were euthanized (n = 8/group, mean ± SEM; Mann-Whitney test, *p < 0.05, **p < 0.01; ns, not significant). (D and F) Survival curves of the vaccinated mice after challenge with either PR8 (D) or Cal07 (F) influenza virus.

proteins with the suboptimal Bn/Bs heterodimerization motif.[26] Thus, the bonus effect of targeting APCs seems to primarily depend on having at least one targeting unit and that adding more targeting units per molecule does not add much. This opens the possibility of constructing heterodimeric vaccine proteins with two different APC-specific targeting moieties, which could result in synergistic effects. For example, combining anti-MHCII[17,22] and anti-CD11c[22] could possibly enhance induction of antibodies, while combining MIP1α[16] and anti-CD40[15] could possibly elicit stronger T cell responses. Such dual targeting specificities within a single heterodimeric vaccine molecule could simultaneously cross-link different receptors on APCs and thus influence intracellular signaling. Alternatively, in a four-armed heterodimeric vaccine protein, only one arm could be used for targeting, leaving three arms for expression of antigens.

APC-targeting heterodimeric DNA vaccines with different antigens on the A and the B chains elicited antibodies and T cells against either antigen. Moreover, all different antigens tested so far have been translated into vaccine proteins irrespective of being expressed on the A or B arm. Thus, the heterodimeric format opens the possibility of broadening the immune response to several different antigens derived from the same pathogen, e.g., HA and neuraminidase from influenza virus. Another possibility is to express different versions of the same antigen, e.g., HA from different influenza viruses, in the same vaccine molecule. Supporting this possibility, introducing a mixture of six different group 1 HAs (H5, H6, H8, H9, H11, H13) into the C_H3-based homodimeric format resulted in protection of immunized mice against a heterosubtypic H1N1 challenge, suggesting that dilution of strain-specific epitopes can result in preferential stimulation of B cells specific for conserved, immunosubdominant epitopes.[34] A recent publication of Kanekiyo et al.[40] using nanoparticles that express eight different H1 HAs support such a dilutional strategy for induction of antibodies against subdominant epitopes.

Finally, utilization of different dimerization motifs should offer the possibility of delivering two distinct APC-targeting DNA vaccines simultaneously. Thus, a single injection of a mix of three plasmids, two plasmids encoding the A and B chains of a heterodimeric molecule and one plasmid encoding a C_H3-based homodimeric molecule, should result in secretion of two distinct vaccine molecules without any mixing between the two (due to the specific interactions between dimerization domains in the endoplasmic reticulum of the transfected cell). One application would be to choose different targeting units for the heterodimeric and the homodimeric vaccine molecules

so that one type of vaccine molecule would primarily induce T cells while the other primarily would elicit antibodies. Alternatively, the same targeting unit could be used in the two types of vaccine molecules while the number of different antigens could be expanded. Possibly, by employing even more dimerization motifs than A/B and C_H3, the above scenario could be further expanded. In summary, the current technology, and its extensions, could increase the versatility of APC-targeting DNA vaccines.

MATERIALS AND METHODS

Ethics Statement

All animal experiments were approved by the Animal Ethics Committee of the National Committee for Animal Experiments (Oslo, Norway) and were carried out in strict accordance with the recommendations in the *Guide for the Care and Use of Laboratory Animals* of the National Institutes of Health.

Mice and Cell Lines

Female BALB/c mice 6–8 weeks of age were purchased from Taconic (Denmark). The $\lambda2^{315}$-specific TCR-transgenic mice on a BALB/c background have been described previously.[41] HEK293E cells were from American Type Culture Collection (ATCC, Manassas, VA, USA), and Esb-MP cells were kindly provided by Dr. J. Van Damme (University of Leuven, Leuven, Belgium). Cells were cultured in RPMI 1640 (Invitrogen) supplemented with 10% heat-inactivated fetal calf serum (FCS, Biochrom), 0.1 mM non-essential amino-acids (Lonza), 1 mM sodium pyruvate (Lonza), 50 μM monothioglycerol (Sigma), and 24 mg/L gentamicin (Sanofi-Aventis Norway) at 37°C with 5% CO_2 in humidified air.

Generation of Heterodimeric DNA Vaccines

All vaccine constructs explored the pLNOH2 vector containing a cytomegalovirus (CMV) promoter and the V_H leader derived from the V_H gene of B1–8.[42] The homodimeric vaccibodies MIP1α-C_H3-scFv[315] and scFv[αNIP]-C_H3-scFv[315] have been described previously.[16] In this vaccine the dimerization motif consists of the hinge exons 1 and 4 and C_H3 from human IgG3. The construction of the Bn/Bs heterodimers has also been described.[26] The cDNAs encoding the ACID (A: AQLEKELQALEKENAQLEKELQALEKELAQ)- and BASE (B: AQLKKKLQALKKKNAQLKKKLQALKKKLAQ)-modified leucine zipper motif were a kind gift from Dr. Elizabeth Mellins.[25] The genes were amplified by PCR. The following primers were used: 5′A/B, 5′-GGAGGTAGCAGCGGTGGAAAATTCGGCGGTTCCACTACA-3′; 3′A, 5′-CCCTCCGCTACCACCTCCCTGAGCCAGTTCCTTTTCCAG-3′; and 3′B, 5′-CCCTCCGCTACCACCTCCCTGGGCGAGTTTCTTCTTGAG-3′. The hinge region of scFv[αNIP]-C_H3-scFv[315] was amplified by the following: 5′h1, 5′-GGTGAGTCGTACGCTAGCAA-3′ and 3′h1, 5′-TCCACCGCTGCTACCTCCTGGGCACCGTGGGCATGTGTGAGTTGTGTCACCAAG-3′. The resulting two PCR products formed the template for a new PCR with 5′h1 primer and 3′A or 3′B primer. The PCR products were digested with *Hind*III and *Sfi*I and subcloned into scFv[αNIP]-C_H3-scFv[315]. Finally, a new PCR was done with primers 5′L2A/B (5′-GGAGGTGGTAGCGGAGGGTTAACCAAATTCGGCGGTTCCACTACA-3′) and the 3′A or 3′B primer. The resulting PCR product was subcloned into *Hind*III and *Sfi*I of scFv[αNIP]-C_H3-scFv[315], generating the heterodimeric A/B vaccine with either two ACID or two BASE motifs connected with the linker GGGSGGLTKFGGSTTAPS. The hinge region upstream of the A/B dimerization motif consists of hinge exon 1 only from the hIgG3, similar to the Bs/Bn heterodimeric vaccine.

The same unique restriction enzyme sites were used for swapping of either the targeting unit (*Bsm*I and *Bsi*WI) or the antigenic unit (*Sfi*I and *Sfi*I) into the three variants of targeting DNA vaccines. Targeting units subcloned N-terminally were MIP1α, the MHCII targeting unit (scFv[αMHCII], from mouse 14-4-4S anti-MHCII mAb specific for the Ia.7 determinant on I-E[d] molecules),[17] and mouse Xcl1,[23] as well as the non-targeting scFv specific for the hapten NIP. Antigenic units used were scFv[315] (M315 myeloma protein) and scFv[A20] (mouse B cell lymphoma A20),[17] HA antigen from influenza A viruses PR8 and Cal07,[32] OVA,[20] and mCherry.[26]

ELISAs for Vaccine Protein Analysis

ELISAs were performed in Costar 96-well plates (Corning Life Sciences) coated with dinitrophenyl (DNP)-BSA (2.5 μg/mL), NIP-BSA (2.5 μg/mL), anti-A/B mAb (1.0 μg/mL, 2H11, a kind gift from Dr. Ellis L. Reinherz),[39] anti-mCherry mAb (1.0 μg/mL),[43] or anti-Cal07 HA mAb (1.0 μg/mL, 29E3, a kind gift from Dr. Thomas Moran). Plates were blocked with PBS containing 1% BSA (25°C for 1 h). 1 μg of DNA per plasmid in 50 μL of Opti-MEM (Life Technologies) and 2 μL of Lipofectamine 2000 (Invitrogen) in 50 μL of Opti-MEM were mixed and incubated for 20 min at 25°C. The DNA/Lipofectamine solution was added to the HEK293E cells (1×10^5 cells/well in a 24-well plates) and incubated at 37°C in a 5% CO_2 humidified atmosphere. Supernatants were collected after 3 days and run in triplicates, serially diluted 3-fold in ELISA buffer (PBS containing 0.2% Tween 20 and 0.1% BSA, 0.02% sodium azide), and incubated for at least 1 h at 25°C. Biotinylated anti-PR8 HA mAb (1 μg/mL, H-36-4-52, a kind gift from Siegfried Weiss), biotinylated anti-Cal07 HA (1.0 μg/mL), biotinylated anti-MIP1α (1 μg/mL, MAB450, R&D Systems), biotinylated anti-scFv[315] (1.0 μg/mL, Ab2-1.4), biotinylated anti-λ (1.0 μg/mL, 9A8 rat anti-mouse Vλ mAb, specific for Vλ1 in scFv[αNIP] and cross-reacting with the Vλ2 domain in scFv[315]), and anti-Xcl1 (1 μg/mL, LS-C16241, LifeSpan BioSciences [LSBio], followed by anti-rabbit IgG-AP [alkaline phosphatase], 1:3,000, from Sigma) were used to detect the respective vaccine constructs (45 min at 25°C). Biotinylated secondary reagents were followed by streptavidin-AP (1:3,000, GE Healthcare) and developed with the addition of phosphatase substrate (1 mg/mL, Sigma-Aldrich) dissolved in diethanolamine substrate buffer. Optical density 405 (OD_{405}) was measured with a Tecan Sunrise microplate reader using the Magellan v5.03 program.

Analysis of the Vaccine Proteins by SDS-PAGE and Western Blot

Vaccine proteins were produced by transient transfection of HEK293E cells as described above, but up-scaled in six-well plates. The DNA vaccines used in this study included a C-terminal histidine (His) tag. Supernatants were collected after 3 days and vaccine

proteins were purified on His SpinTrap columns (GE Healthcare, Buckinghamshire, UK). The vaccine proteins were analyzed by SDS-PAGE using 4%–12% Novex Tris-glycine gels (Invitrogen) and detected with biotinylated anti-MIP1α or anti-λ1 (9A8, specific for λ1) and streptavidin-HRP (horseradish peroxidase, 1:10,000, GE Healthcare) for 1 h at 25°C before being developed with an enhanced chemiluminescence (ECL) Advance western blotting detection kit (GE Healthcare) in a G:Box Chemi XX6 device (Syngene). The Spectra multicolor broad range protein ladder (Life Technologies) was used to determine the sizes of the vaccine proteins.

Chemotaxis Assay

600 μL of medium (RPMI 1640 with 1% BSA) containing recombinant MIP1α (0.05 μg/mL, positive control, PeproTech) or vaccine proteins (supernatants harvested from transfected HEK293E, 4-fold diluted) was added to the bottom wells of Transwell plates (5-μm-pore polycarbonate membrane). 100 μL (2×10^6) of $CCR1^+CCR5^+$ ESb-MP cells was added to the upper wells and incubated 2 h at 37°C. Cells that migrated to the bottom wells were counted by a Countess automated cell counter (Invitrogen). The chemotactic index was calculated as fold increase in the number of cells migrating in the presence of chemotactic factors by the various vaccine proteins as compared to spontaneous cell migration (medium alone or mock).

DNA Vaccination and EP

Mice were anesthetized by an intraperitoneal (i.p.) injection with a mixture of Zoletil forte (250 mg/mL), xylazine (Rompun; 20 mg/mL), and fentanyl (50 μg/mL) (ZRF, 6 mL/kg body weight) before each hind leg was shaved to prepare for vaccination. Plasmid DNA was purified using an EndoFree Mega plasmid purification system (-QIAGEN). 50 μL of 0.5 μg/μL DNA per plasmid dissolved in sterile 0.9% saline solution (B. Braun) was injected i.m. into each *quadriceps femoris* muscle. The amount was 50 μg of DNA per plasmid per mouse, unless otherwise indicated in the figure legends. The plasmid sizes ranged from 8,783 bp (the $scFv^{\alpha MHCII}$-C_H3-HA plasmid) to 7,351 bp (the MIP1α-A-$scFv^{315}$ plasmid). EP was performed immediately after injection through the delivery of pulses from electrodes inserted i.m. flanking the injection site (Needle EP) using an Elgen electroporator device from Inovio Biomedical, as described.[22] For intradermal vaccination, the lower back was shaved. 25 μL of 0.5 μg/μL DNA per plasmid was injected into each flank of the lower back, followed by EP using DermaVax (BTX/Harvard Apparatus), as previously described.[32] Blood samples were collected from the saphenous vein at regular intervals, spun down twice, and sera were frozen at −20°C.

Tumor Challenge

MOPC315.4, MOPC315.BM, and MOPC315.BM.Luc cells were cultured *in vitro* (37°C, 5% CO_2) and harvested during the exponential growth phase, then centrifuged (300 × *g*, 7 min) and resuspended in PBS. Mice were vaccinated once as described above and challenged with tumor cells 14 days later as follows: (1) s.c. on the right flank with 10^5 MOPC.315.4 myeloma tumor cells (n = 10/group). The size of the tumor was followed. A tumor diameter of 10 mm was defined as a humane endpoint, at which time mice were euthanized. (2) i.v. with 10^4 MOPC.315.BM cells (n = 10/group).[29] Mice reaching endpoint paraplegia were euthanized. (3) i.v. with 2×10^5 MOPC.315.BM.Luc cells (n = 4–8/group). In the experiment with MOPC.315.BM/Luc, from day 12 mice were injected every other day for 14 days and after that once a week with 100 μg of depleting mAb against CD8 (TIB-105, ATCC) or with isotype control Ab (Y13-238, ATCC). Imaging of Luc activity *in vivo* was performed essentially as described.[29] Mice were injected i.p. with D-luciferin (150 mg/kg body weight) and imaged using the IVIS Spectrum imaging systems (Caliper Life Sciences, Hopkinton, MA, USA). Images were acquired from 10 to 20 min after substrate injection. Data were analyzed using Living-Image software (Caliper Life Sciences). Each image included a non-tumor-bearing control mouse also administered D-luciferin. Luminescence from each side or region of tumor-bearing mice was quantified using average photons/s/cm^2/steradian, with the respective area on the non-tumor-bearing control mice subtracted. Disease was verified by measurement of M315 myeloma protein in ELISA.[28]

IFN-γ ELISPOT

Adoptively transferred BALB/c mice (3×10^6 $\lambda2^{315}$-TCR-transgenic $CD4^+$ T cells/mouse) were vaccinated the next day with 50 μg of DNA per plasmid i.m. as described above. 11 days after vaccination, spleens were harvested and splenocytes prepared using the gentleMACS dissociator (Miltenyi Biotec) according to the manufacturer's enzyme-free protocol. Splenic IFN-γ peptide-specific T cells were determined by an $ELIspot^{PLUS}$ for mouse IFN-γ kit according to the manufacturer's protocol (Mabtech, Nack Straand, Sweden). Briefly, splenocytes were dissociated and treated with Tris-buffered ammonium chloride (ACT) for 7 min on ice, washed three times with RPMI 1640, and filtered through a 70-μm nylon strainer to prepare single-cell suspensions. Splenocytes were plated in triplicates (1×10^6, 5×10^5, and 2.5×10^5 cells/well) and synthetic peptide (5.0 μg/mL, ALWFRNHFVFGGGTKVT, amino acids 91–107 of $\lambda2^{315}$ Ig L chain) or irrelevant control peptide (5.0 μg/mL, HNTNGVTAACSHEG from HA) was added.

BALB/c mice were vaccinated i.d. with 25 μg of DNA per plasmid. Spleens were harvested after 14 days, plated in triplicates (5×10^5 cells/well), and stimulated with 2 μg/mL MHCII (SVSSFERFEIFPK and HNTNGVTAACSHEG)- or MHCI (IYSTVASSL, both InvivoGen)-restricted HA^{PR8} peptides, MHCII-restricted OVA peptide (amino acids 323–339, ISQAVHAAHAEINEAGR, InvivoGen), or complete recombinant HA^{PR8} protein (2 μg/mL, Sino Biological) or OVA protein (5 μg/mL, InvivoGen). Spots were counted electronically and analyzed using the CT ELISPOT reader (CTL Europe, Bonn, Germany).

Influenza Challenge of Vaccinated Mice

Vaccinated mice where challenged after a single vaccination i.m. as described above. Groups of anesthetized mice were infected intranasally with 7.5 × lethal dose 50 (LD_{50}) of PR8 or 5 × LD_{50} of Cal07 influenza viruses in 20 μL of PBS (10 μL per nostril PR8 or Cal07, kindly provided by Dr. Anna Germundsson Hauge at the National

Veterinary Institute, Oslo, Norway). LD_{50}s of PR8 and Cal07 were determined as described before.[32] Mice were monitored for weight loss, and the humane endpoint was >20% weight loss, as required by the Norwegian Animal Research Authority.

ELISA for Detection of Antigen-Specific Antibodies in Serum from BALB/c Mice

High-binding 96-well ELISA plates (Costar) were coated with 2 μg/mL myeloma protein M315 (IgA, λ2) or recombinant HA (PR8, 0.5 μg/mL, or Cal07, 1 μg/mL, both Sino Biological). Serum samples were serially diluted 3-fold, starting at 1:50 in ELISA buffer. Abs in sera were detected with AP-conjugated anti-IgG (1:3,000, Sigma-Aldrich), biotinylated anti-IgG1a (1.0 μg/mL, BD Pharmingen), or anti-IgG2a (1.0 μg/mL, BD Pharmingen) and incubated for 1 h at 25°C. Otherwise, the serum ELISAs were run as described above for protein vaccine ELISA. Antibody titer was defined as the highest dilution of a serum sample with OD values greater than the mean × 2 of saline-vaccinated mice. Samples with a titer <50 were given an endpoint titer of 1.

Statistical Analysis

All statistical analyses were performed using the GraphPad Prism version 7.04. Statistical differences between antibody titers and luminescence curves at various time points were compared with two-way ANOVA. Differences in survival were calculated by Mantel-Cox analysis. Differences in tumor-specific antigen M315, M315- or HA-specific total IgG, IgG1, and IgG2a, and weight curves after influenza challenge were calculated using a two-tailed Mann-Whitney test. A one-tailed Mann-Whitney test was used for the IFN-γ T cell ELISPOT. A p value of 0.05 or less was considered statistically significant.

SUPPLEMENTAL INFORMATION

Supplemental Information can be found online at https://doi.org/10.1016/j.omtm.2020.01.007.

AUTHOR CONTRIBUTIONS

R.B., H.C.L.S., and B.B. conceived the idea and designed the experiments. R.B., H.C.L.S., D.M.H., J.B., S.B., and E.F. performed the experiments. R.B. and B.B. wrote the paper, with contributions from H.C.L.S. and D.M.H. All authors reviewed the manuscript.

CONFLICTS OF INTEREST

The TTO office of the University of Oslo (UoO), Inven2, has filed a provisional patent application no. 62/555,305 "Vaccine molecules" on A/B heterodimeric vaccine molecules. UoO employees R.B. and B.B. are inventors. The remaining authors declare no competing interests.

ACKNOWLEDGMENTS

We thank Dr. Elizabeth Mellins (Stanford University Medical Center, Stanford, CA, USA) for providing vectors encoding the ACID and BASE motifs, and Dr. Ellis L. Reinherz (Dana-Farber Cancer Institute, Boston, MA, USA) for the anti-A/B mAb. We gratefully acknowledge Elisabeth Vikse, Hilde Omholt, and Peter Hofgaard for expert assistance. The study was supported by grants from Research Council of Norway, Norway, NanoCanTher; INDIGO-IPP2-060/Inno Indigo project 261091 (to B.B.); South-Eastern Norway Regional Health Authority, Norway, project Innovation 2016/2017 (to B.B.); South-Eastern Norway Regional Health Authority, Norway, project 2019127 (to R.B.); the K.G. Jebsen Centre for Influenza Vaccine Research, Norway (to B.B.); and by Norwegian Cancer Society, Norway, project 182354 (to B.B.).

REFERENCES

1. Davis, B.S., Chang, G.J., Cropp, B., Roehrig, J.T., Martin, D.A., Mitchell, C.J., Bowen, R., and Bunning, M.L. (2001). West Nile virus recombinant DNA vaccine protects mouse and horse from virus challenge and expresses in vitro a noninfectious recombinant antigen that can be used in enzyme-linked immunosorbent assays. J. Virol. *75*, 4040–4047.
2. Bergman, P.J., McKnight, J., Novosad, A., Charney, S., Farrelly, J., Craft, D., Wulderk, M., Jeffers, Y., Sadelain, M., Hohenhaus, A.E., et al. (2003). Long-term survival of dogs with advanced malignant melanoma after DNA vaccination with xenogeneic human tyrosinase: a phase I trial. Clin. Cancer Res. *9*, 1284–1290.
3. Salonius, K., Simard, N., Harland, R., and Ulmer, J.B. (2007). The road to licensure of a DNA vaccine. Curr. Opin. Investig. Drugs *8*, 635–641.
4. Trimble, C.L., Morrow, M.P., Kraynyak, K.A., Shen, X., Dallas, M., Yan, J., Edwards, L., Parker, R.L., Denny, L., Giffear, M., et al. (2015). Safety, efficacy, and immunogenicity of VGX-3100, a therapeutic synthetic DNA vaccine targeting human papillomavirus 16 and 18 E6 and E7 proteins for cervical intraepithelial neoplasia 2/3: a randomised, double-blind, placebo-controlled phase 2b trial. Lancet *386*, 2078–2088.
5. Lee, S.H., Danishmalik, S.N., and Sin, J.I. (2015). DNA vaccines, electroporation and their applications in cancer treatment. Hum. Vaccin. Immunother. *11*, 1889–1900.
6. Liu, M.A. (2011). DNA vaccines: an historical perspective and view to the future. Immunol. Rev. *239*, 62–84.
7. Aihara, H., and Miyazaki, J. (1998). Gene transfer into muscle by electroporation in vivo. Nat. Biotechnol. *16*, 867–870.
8. Grodeland, G., Fredriksen, A.B., Løset, G.A., Vikse, E., Fugger, L., and Bogen, B. (2016). Antigen targeting to human HLA class II molecules increases efficacy of DNA vaccination. J. Immunol. *197*, 3575–3585.
9. Snider, D.P., and Segal, D.M. (1987). Targeted antigen presentation using crosslinked antibody heteroaggregates. J. Immunol. *139*, 1609–1616.
10. Kawamura, H., and Berzofsky, J.A. (1986). Enhancement of antigenic potency in vitro and immunogenicity in vivo by coupling the antigen to anti-immunoglobulin. J. Immunol. *136*, 58–65.
11. Castro, F.V., Tutt, A.L., White, A.L., Teeling, J.L., James, S., French, R.R., and Glennie, M.J. (2008). CD11c provides an effective immunotarget for the generation of both CD4 and CD8 T cell responses. Eur. J. Immunol. *38*, 2263–2273.
12. Baier, G., Baier-Bitterlich, G., Looney, D.J., and Altman, A. (1995). Immunogenic targeting of recombinant peptide vaccines to human antigen-presenting cells by chimeric anti-HLA-DR and anti-surface immunoglobulin D antibody Fab fragments in vitro. J. Virol. *69*, 2357–2365.
13. Lunde, E., Munthe, L.A., Vabø, A., Sandlie, I., and Bogen, B. (1999). Antibodies engineered with IgD specificity efficiently deliver integrated T-cell epitopes for antigen presentation by B cells. Nat. Biotechnol. *17*, 670–675.
14. Nchinda, G., Kuroiwa, J., Oks, M., Trumpfheller, C., Park, C.G., Huang, Y., Hannaman, D., Schlesinger, S.J., Mizenina, O., Nussenzweig, M.C., et al. (2008). The efficacy of DNA vaccination is enhanced in mice by targeting the encoded protein to dendritic cells. J. Clin. Invest. *118*, 1427–1436.
15. Schjetne, K.W., Fredriksen, A.B., and Bogen, B. (2007). Delivery of antigen to CD40 induces protective immune responses against tumors. J. Immunol. *178*, 4169–4176.
16. Fredriksen, A.B., and Bogen, B. (2007). Chemokine-idiotype fusion DNA vaccines are potentiated by bivalency and xenogeneic sequences. Blood *110*, 1797–1805.

17. Fredriksen, A.B., Sandlie, I., and Bogen, B. (2006). DNA vaccines increase immunogenicity of idiotypic tumor antigen by targeting novel fusion proteins to antigen-presenting cells. Mol. Ther. *13*, 776–785.

18. Demangel, C., Zhou, J., Choo, A.B., Shoebridge, G., Halliday, G.M., and Britton, W.J. (2005). Single chain antibody fragments for the selective targeting of antigens to dendritic cells. Mol. Immunol. *42*, 979–985.

19. Ruffini, P.A., Biragyn, A., Coscia, M., Harvey, L.K., Cha, S.C., Bogen, B., and Kwak, L.W. (2004). Genetic fusions with viral chemokines target delivery of nonimmunogenic antigen to trigger antitumor immunity independent of chemotaxis. J. Leukoc. Biol. *76*, 77–85.

20. Grødeland, G., Mjaaland, S., Tunheim, G., Fredriksen, A.B., and Bogen, B. (2013). The specificity of targeted vaccines for APC surface molecules influences the immune response phenotype. PLoS ONE *8*, e80008.

21. Grødeland, G., Fossum, E., and Bogen, B. (2015). Polarizing T and B cell responses by APC-targeted subunit vaccines. Front. Immunol. *6*, 367.

22. Braathen, R., Spång, H.C.L., Lindeberg, M.M., Fossum, E., Grødeland, G., Fredriksen, A.B., and Bogen, B. (2018). The magnitude and IgG subclass of antibodies elicited by targeted DNA vaccines are influenced by specificity for APC surface molecules. Immunohorizons *2*, 38–53.

23. Fossum, E., Grødeland, G., Terhorst, D., Tveita, A.A., Vikse, E., Mjaaland, S., Henri, S., Malissen, B., and Bogen, B. (2015). Vaccine molecules targeting Xcr1 on cross-presenting DCs induce protective $CD8^+$ T-cell responses against influenza virus. Eur. J. Immunol. *45*, 624–635.

24. Gudjonsson, A., Lysén, A., Balan, S., Sundvold-Gjerstad, V., Arnold-Schrauf, C., Richter, L., Bækkevold, E.S., Dalod, M., Bogen, B., and Fossum, E. (2017). Targeting influenza virus hemagglutinin to $Xcr1^+$ dendritic cells in the absence of receptor-mediated endocytosis enhances protective antibody responses. J. Immunol. *198*, 2785–2795.

25. Busch, R., Pashine, A., Garcia, K.C., and Mellins, E.D. (2002). Stabilization of soluble, low-affinity HLA-DM/HLA-DR1 complexes by leucine zippers. J. Immunol. Methods *263*, 111–121.

26. Spång, H.C., Braathen, R., and Bogen, B. (2012). Heterodimeric barnase-barstar vaccine molecules: influence of one versus two targeting units specific for antigen presenting cells. PLoS ONE *7*, e45393.

27. Lynch, R.G., Graff, R.J., Sirisinha, S., Simms, E.S., and Eisen, H.N. (1972). Myeloma proteins as tumor-specific transplantation antigens. Proc. Natl. Acad. Sci. USA *69*, 1540–1544.

28. Lauritzsen, G.F., Weiss, S., Dembic, Z., and Bogen, B. (1994). Naive idiotype-specific $CD4^+$ T cells and immunosurveillance of B-cell tumors. Proc. Natl. Acad. Sci. USA *91*, 5700–5704.

29. Hofgaard, P.O., Jodal, H.C., Bommert, K., Huard, B., Caers, J., Carlsen, H., Schwarzer, R., Schünemann, N., Jundt, F., Lindeberg, M.M., and Bogen, B. (2012). A novel mouse model for multiple myeloma (MOPC315.BM) that allows noninvasive spatiotemporal detection of osteolytic disease. PLoS ONE *7*, e51892.

30. Bogen, B., Munthe, L., Sollien, A., Hofgaard, P., Omholt, H., Dagnaes, F., Dembic, Z., and Lauritzsen, G.F. (1995). Naive $CD4^+$ T cells confer idiotype-specific tumor resistance in the absence of antibodies. Eur. J. Immunol. *25*, 3079–3086.

31. Snodgrass, H.R., Fisher, A.M., Bruyns, E., and Bogen, B. (1992). Restricted α/β receptor gene usage of idiotype-specific major histocompatibility complex-restricted T cells: selection for CDR3-related sequences. Eur. J. Immunol. *22*, 2169–2172.

32. Grodeland, G., Mjaaland, S., Roux, K.H., Fredriksen, A.B., and Bogen, B. (2013). DNA vaccine that targets hemagglutinin to MHC class II molecules rapidly induces antibody-mediated protection against influenza. J. Immunol. *191*, 3221–3231.

33. Lysén, A., Braathen, R., Gudjonsson, A., Tesfaye, D.Y., Bogen, B., and Fossum, E. (2019). Dendritic cell targeted Ccl3- and Xcl1-fusion DNA vaccines differ in induced immune responses and optimal delivery site. Sci. Rep. *9*, 1820.

34. Anderson, A.M., Baranowska-Hustad, M., Braathen, R., Grodeland, G., and Bogen, B. (2018). Simultaneous targeting of multiple hemagglutinins to APCs for induction of broad immunity against influenza. J. Immunol. *200*, 2057–2066.

35. Løvås, T.O., Bruusgaard, J.C., Øynebråten, I., Gundersen, K., and Bogen, B. (2014). DNA vaccines: MHC II-targeted vaccine protein produced by transfected muscle fibres induces a local inflammatory cell infiltrate in mice. PLoS ONE *9*, e108069.

36. Roos, A.K., Eriksson, F., Timmons, J.A., Gerhardt, J., Nyman, U., Gudmundsdotter, L., Bråve, A., Wahren, B., and Pisa, P. (2009). Skin electroporation: effects on transgene expression, DNA persistence and local tissue environment. PLoS ONE *4*, e7226.

37. Horlick, R.A., Schilling, A.E., Samama, P., Swanson, R.N., Fitzpatrick, V.D., Robbins, A.K., and Damaj, B. (2000). Combinatorial gene expression using multiple episomal vectors. Gene *243*, 187–194.

38. O'Shea, E.K., Lumb, K.J., and Kim, P.S. (1993). Peptide "Velcro": design of a heterodimeric coiled coil. Curr. Biol. *3*, 658–667.

39. Chang, H.C., Bao, Z., Yao, Y., Tse, A.G., Goyarts, E.C., Madsen, M., Kawasaki, E., Brauer, P.P., Sacchettini, J.C., Nathenson, S.G., et al. (1994). A general method for facilitating heterodimeric pairing between two proteins: application to expression of alpha and beta T-cell receptor extracellular segments. Proc. Natl. Acad. Sci. USA *91*, 11408–11412.

40. Kanekiyo, M., Joyce, M.G., Gillespie, R.A., Gallagher, J.R., Andrews, S.F., Yassine, H.M., Wheatley, A.K., Fisher, B.E., Ambrozak, D.R., Creanga, A., et al. (2019). Mosaic nanoparticle display of diverse influenza virus hemagglutinins elicits broad B cell responses. Nat. Immunol. *20*, 362–372.

41. Bogen, B., Gleditsch, L., Weiss, S., and Dembic, Z. (1992). Weak positive selection of transgenic T cell receptor-bearing thymocytes: importance of major histocompatibility complex class II, T cell receptor and CD4 surface molecule densities. Eur. J. Immunol. *22*, 703–709.

42. Norderhaug, L., Olafsen, T., Michaelsen, T.E., and Sandlie, I. (1997). Versatile vectors for transient and stable expression of recombinant antibody molecules in mammalian cells. J. Immunol. Methods *204*, 77–87.

43. Øynebråten, I., Løvås, T.O., Thompson, K., and Bogen, B. (2012). Generation of antibody-producing hybridomas following one single immunization with a targeted DNA vaccine. Scand. J. Immunol. *75*, 379–388.

OMTM, Volume *17*

Supplemental Information

A DNA Vaccine That Encodes an Antigen-Presenting Cell-Specific Heterodimeric Protein Protects against Cancer and Influenza

Ranveig Braathen, Heidi Cecilie Larsen Spång, Daniëla Maria Hinke, Jana Blazevski, Sonja Bobic, Even Fossum, and Bjarne Bogen

Supplemental information

Supplemental Figures

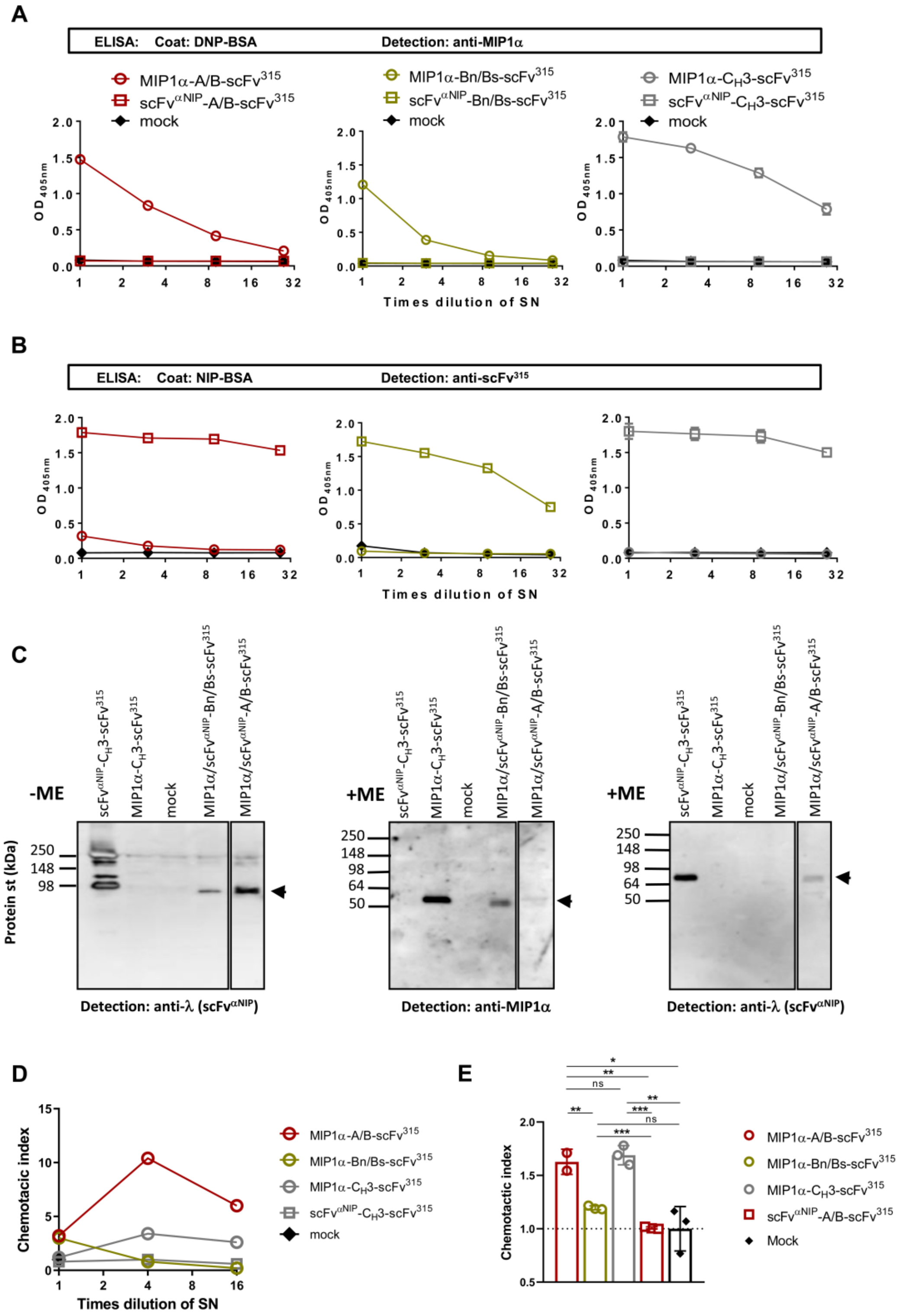

Figure S1. Characterisation of heterodimeric and homodimeric APC-targeting DNA vaccines. A-B and D. Supernatants from HEK293E cells transiently transfected with the indicated combinations of DNA plasmids were analyzed in the specified sandwich ELISAs (mean ±SD) (A-B) and for chemotaxis of $CCR1^+$ $CCR5^+$ ESb-MP cells (D). **C.** Western analysis of homodimeric and heterodimeric APC-targeting DNA vaccines. Western analysis were performed on supernatants from HEK293E cells transiently transfected with the indicated combinations of DNA plasmids containing a HIS tag. The supernatants were purified on a HIS-column before adding to the gel under non-reducing (-ME) or reducing (+ME) conditions and vaccine proteins detected as specified. An arrowhead shows the protein band detected by the specific antibodies against λ-chain in $scFv^{315}$ or MIP1α, respectively. The vertical lines indicate where parts of the image were joined. **E.** Chemotaxis on DC-enriched bone marrow cells from BALB/c mice was performed with 40 nM of the indicated affinity purified vaccine proteins (n=3 filter/vaccine group, n=2 for MIP1α-A/B-$scFv^{315}$, mean±SD, ns=non-significant, *p<0.05, **p<0.01 ***p<0.001, Unpaired T test).

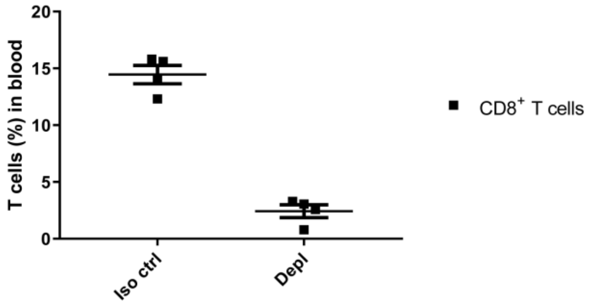

Figure S2. Depletion of CD8^{+} T cells by injection of anti-CD8 mAb. Mice were immunized with 50 µg i.m./EP of MIP1α-A/B-scFv^{315} DNA vaccine. From day 12 mice were injected every other day for 14 days and after that once a week with 100 µg of depleting mAb against CD8 or with isotype control Ab. 2x10^{5} MOPC315.BM.Luc tumor cells were used i.v. as challenge on day 14 after vaccination. Amount of T cells (%) in peripheral blood at day 38 after challenge in the specified group (n=4/group).

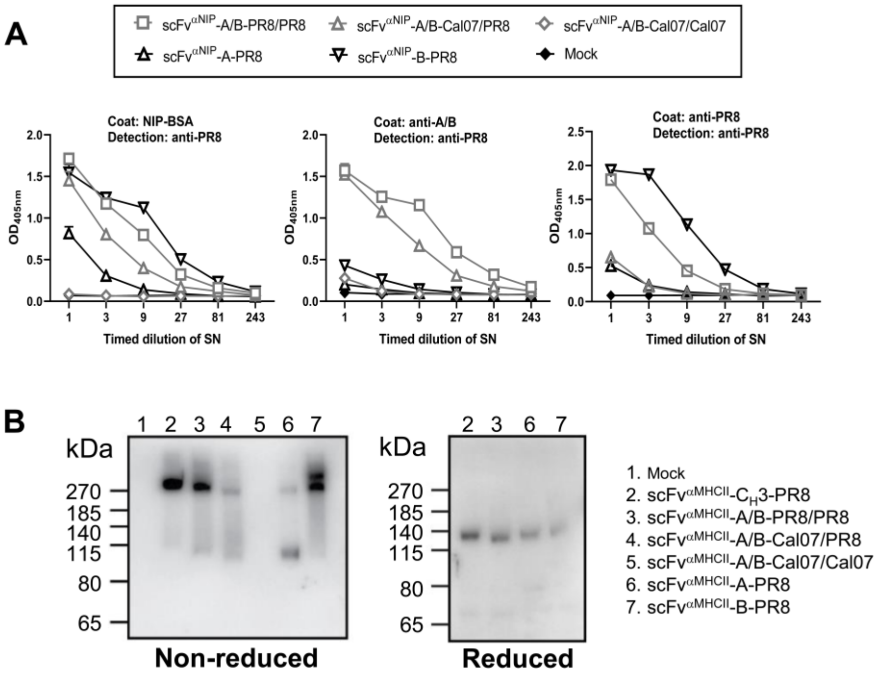

Figure S3. Both the ACID and BASE chains alone can be expressed in vitro. A. HEK293E cells were transiently transfected with a total amount of 5 μg of DNA per transfection with the indicated combinations of DNA plasmids. Supernatants were analyzed in the indicated sandwich ELISAs (mean of triplicates ±SD). The graph to the right indicates preferential A/B pairing since the signal caused by B/B dimer formation (PR8 on both arms, detected in the anti-PR8/anti PR8 sandwich ELISA) was attenuated by inclusion of an A-chain with a different antigen (Cal07 HA). **B**. Western blot analysis of supernatants. Supernatants were first normalized on dot blots. Adjusted amounts were loaded onto the gel under non-reducing (-DTT) or reducing (+0.1 M DTT) conditions. Vaccine proteins were detected with anti-PR8 specific mAb.

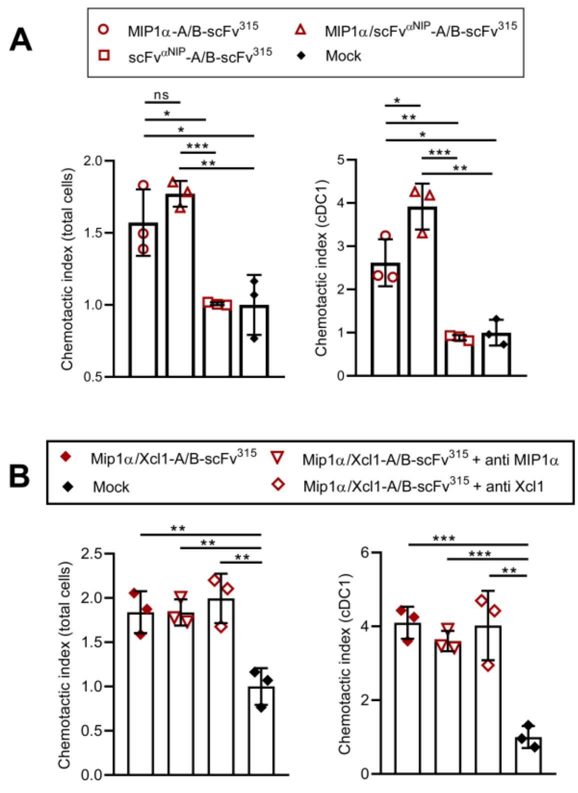

Figure S4. A single APC-specific targeting unit in the ACID/BASE heterodimeric vaccine is sufficient for chemotaxis of cDC1 cells. A. Chemotaxis on Flt3L induced DC-enriched bone marrow cells from BALB/c mice was performed with 40 nM of the indicated purified vaccine proteins. The chemotaxis index was measured by flow and shown for either total number of cells (left) or cDC1 ($CD11c^{+}CD45^{-}CD11b^{-}CD24^{+}$) cells (right). **B.** Purified vaccine protein (2 nM), with MIP1α chemokine on the A arm and Xcl-1 chemokine (a known cDC1 attractant) on the B arm, were tested for their ability to attract total cells (left) or cDC1 (right) in the presence of either no mAb, or anti-MIP1α mAb or anti-Xcl1 mAb in 40-fold molar excess (n=3 filter/vaccine group, mean±SD, ns=non-significant, **$p<0.01$ ***$p<0.001$, Unpaired T test).

Supplemental Methods

T cells in peripheral blood

Blood samples (50 µl) were collected from the saphenous vein into a tube precoated with 50µl of 1:10 Heparin: H20 solution (Heparin 5000 IE/ml). Cells were centrifuged (1400 rpm, 7min, 4°C) and incubated with ACT buffer on ice 5-10 min and washed 3x PBS+0.5% BSA. Cells were blocked for unspecific binding by the use of 30% rat serum for 20 min at 4°C. Subsequent staining was done with following antibodies, according to manufacturer`s recommendations: Anti-Mouse CD19- FITC (35-0193-U500, TONBO); Anti-Mouse CD14-FITC (553739, BD Pharmingen); Anti-Mouse CD3-VF450 (75-0032-U100, TONBO); Anti-Mouse CD8-AF700 (100730, Biolegend). The cells were analyzed on the Attune Nxt acoustic focusing cytometer (Life Technologies), using FlowJo software. Cells of interest were gated as $CD19^{-}CD14^{-}CD3^{+}CD8^{+}$.

Purification of vaccine proteins

For purification of $scFv^{315}$ containing vaccine proteins, $2x10^{7}$ HEK293E cells were seeded out in a BD Falcon® 5 layer multilayer flask (Corning), and transfected with a total of 250 µg plasmid vectors (125 + 125 µg for A/B and Bn/Bs vaccine proteins, and 250 for C_H3 vaccine proteins) using 500 µg Polyethylenimine. Vaccine proteins were purified from HEK293E supernatant using a column with DNP (dinitrophenyl)-lysine-Sepharose (Sigma). The column was washed with PBS and 0,05 M hapten analogue N-carbobenzoxy (CBZ)-glycine (pH 7.4, Merck) in 0.5 M Saline. Vaccine proteins were eluted with 0.05 M DNP-glycine (pH 7.3, Sigma) in PBS, and run through a Dowex 1x8-100 Cl form ion-exchange resin (Sigma) to remove the DNP hapten. The concentration of purified proteins was verified by ELISA using 1 µg/ml DNP-BSA as coat and detected with 1 µg/ml biotinylated anti-λ mAb (9A8) or anti-

MIP1α mAb (MAB450), and 1 µg/ml anti-A/B (2H11) as coat and detected with 1 µg/ml biotinylated anti-scFv[315] mAb (Ab2-1.4).

DC-enriched bone marrow cells and chemotaxis assays

For preparation of Flt3L-induced DCs, bone marrow cells were harvested by flushing tibiae and femurs with medium. The cell suspension was filtered through a 70 µm Nylon cell strainer and seeded at a concentration of 2×10^6 cells per ml, 5 ml per well in a six-well plate. Then 0.1 µg/ml of Flt3L (Peprotech, NJ) was added and the cells were incubated for 9 d at 37°C 5% CO_2. Semiadherent cells were then harvested and used for chemotaxis. 200 µl medium (RPMI1640 with 1% bovine serum albumin) containing various purified vaccine proteins were added to the bottom wells of Transwell plates (5 µm pore polycarbonate membrane). 200 µl ($2x10^6$) Flt3L-induced DC cells were added to the upper wells and incubated 4 hours at 37°C. Cells that migrated to the bottom wells were analyzed by flow cytometry. The cDC1 population was gated based on staining with anti-CD45R/B220 (RA3-6B2; Tonbo Biosciences), anti-CD11c (N418; Tonbo Biosciences), anti-CD11b (M1/70; Tonbo Biosciences) and anti-CD24 (M1/69; BioLegend). In some of the chemotaxis experiments, 2 µg of either anti-MIP1α mAb (MAB450, R&D Systems) or anti-Xcl1 mAb (LS-C16241, Lifespan Biosciences) were added together with the purified protein (2 nM or 0.05 µg). The chemotactic index was calculated as fold increase in the number of cells migrating in the presence of chemotactic factors by the various vaccine proteins as compared to spontaneous cell migration (medium alone or mock).
